# Comparation of time-course, dose-effect, influencing factors and adverse events of biologics in the treatment of adults with moderate to severe plaque psoriasis

**DOI:** 10.3389/fimmu.2023.1151977

**Published:** 2023-05-25

**Authors:** Weiqi Gao, Boran Yu, Ya Yan, Libo Zhao, Rongsheng Zhao

**Affiliations:** ^1^ Department of Pharmacy, Peking University Third Hospital, Beijing, China; ^2^ Shanxi Bethune Hospital, Shanxi Academy of Medical Sciences, Tongji Shanxi Hospital, Third Hospital of Shanxi Medical University, Taiyuan, China; ^3^ Tongji Hospital, Tongji Medical College, Huazhong University of Science and Technology, Wuhan, China; ^4^ Department of pharmacy, Beijing Children’s Hospital, Capital Medical University, National Center for Children’s Health, Beijing, China; ^5^ School of Pharmacy, Shanxi Medical University, Taiyuan, China; ^6^ Institute for Drug Evaluation, Peking University Health Science Center, Beijing, China; ^7^ Therapeutic Drug Monitoring and Clinical Toxicology Center, Peking University, Beijing, China

**Keywords:** model-based meta-analysis, biologics, moderate to severe psoriasis, relative efficacy, adverse events comparation

## Abstract

**Introduction:**

Biologics is used for treating moderate to severe plaque psoriasis (MSPP), which represent one of the foremost therapeutic advancements in disease of dermatology. Up to now, the relative efficacy and safety across approved andinvestigational biologics for MSPP is still unclear.

**Methods:**

This study aimed to comparative effectiveness of various biological treatments for MSPP measured by PASI75, PASI90 and PASI100 (The ratio of patients whose Psoriasis Area and Severity Index score (PASI) decreased by ≥ 75%, 90% and 100% compared with baseline, respectively). In addition, random models were used together with a Bayesian method to compare direct and indirect Adverse Events (AEs) of biologics with placebo, to make probabilistic statements and predictions on their AEs. The analytic data set was made up of summarized data from 54 trials, including 27,808 patients, with treatment of 17 biologics. Three mathematic models with nonparametric placebo evaluations were established to characterize the longitudinal direction profile for the three efficacy measures as above mentioned.

**Results:**

Our results showed significant differences among treatments. Bimekizumab, sonelokimab, and ixekizumab were found to be the most effective treatments among the biologics. The effects of covariate were further evaluated, patients’ age, body weight, duration of disease and percentage of patients previously treated with a biological therapy showed impact on the efficacy. In addition, we found that ixekizumab and risankizumab displayed relatively stable as for efficacy and safety.

**Discussion:**

Our findings provide valuable insights into the comparative effectiveness and safety of biologics for MSPP treatment. These results may aid in clinical decision-making and ultimately improve patient outcomes.

## Introduction

Psoriasis, which is a chronic inflammatory disease accompanies systemic involvement characterized by typical cutaneous manifestation, affects 1.5-4.0% of the world’s population. Plaque psoriasis, the most common variation of psoriasis, occurring in more than 80% population with psoriasis (1, 2), which can be divided into three categories containing mild, moderate, or severe, with overlap between these degrees (e.g., mild to moderate and moderate to severe) (3). The current therapeutic options for psoriasis including topical creams, oral agents, phototherapy, and biologics (4). On the whole, biologics is more effective compared to oral drugs or phototherapy (5). The 4 classes of biologics are approved by the US Food and Drug Administration (FDA) to treat psoriasis, which contains inhibitors of tumor necrosis factor (TNF)-α, interleukin (IL)-17, interleukin (IL)-23 and interleukin (IL)-12/23 (6, 7).

Huan He et al’ s research (8) showed a quantified efficacy comparison of 13 biologics for psoriasis according to efficacy. However, over the last two years, there has been rapid development of novel biologics, including bimekizumab, sonelokimab, vunakizumab and so on, which can inhibit one of the IL-17A and IL-17F ligands or both of them for treating MSPP (9). Hence, this article aims to provide latest knowledge about biological effects focusing on MSPP to clinicians. At the same time, clinical randomized controlled trials (RCTs) generally report Adverse Events (AEs) at the end of the induction phase. Thus, the Bayesian approach offers the opportunity to make indirect comparisons among safety outcomes that have not yet been directly compared through trials and provides a more direct way to make probabilistic statements and predictions about their AEs at the end of the induction phase. The second purpose of this study is to rank the AEs of the above biologics at the end of induction phase.

## Methods

### Data source and handling

RCTs included were identified through a systematic literature review (SLR) according to the guidelines and recommendations of the Cochrane Collaboration (10). Researchers performed an electronic literature search in PubMed, Cochrane, EMBASE, and ClinicalTrials.gov website and the start and cutoff date of retrieval were from the creation of database to April 29, 2022. The search keywords were as follows: adalimumab, brodalumab, bimekizumab, briakinumab, certolizumab, etanercept, guselkumab, infliximab, ixekizumab, itolizumab, LY3471851, mirikizumab, risankizumab, secukinumab, sonelokimab, tildrakizumab, ustekinumab, vunakizumab, moderate to severe plaque psoriasis, biologics, and randomized controlled trial.

All trials had to match the following criteria:

1. Trials including adult patients who were diagnosed with MSPP and treated with biologics;
2. Double-blinded randomized placebo controlled or active-controlled trials and published in English;
3. Trials reported at least one of the following outcomes: PASI75, PASI90, PASI100 or AEs.

Results of the initial search were screened, as well as data were extracted independently by Weiqi Gao, Ya Yan, with disagreements settled by other reviewers (Libo Zhao and Rongsheng Zhao). Only data from the induction period of trials were included in analyses. For each eligible trials, the following were the items of the extracted data: title, author, trial name, trial design, publication year, clinical factors (such as percentage of males, age, weight, disease duration, percentage of patients previously treated with a biological therapy and baseline PASI) and primary outcome. Outcomes of efficacy and safety were extracted from tables, figures and text, containing PASI75, PASI90, PASI100 and AEs. Researchers normalized different dose schemes to collect the same drug efficacy data. Dosing for weight-based dosing regimens was normalized to 70 kg.

The intent-to-treat populations were used whenever available in the development of the analytic dataset. The specific inclusion criteria of covariates in model analysis were as follows: In the analytic dataset, if the missing value of a covariate was ≤ 40%, it will be incorporated into the final models and the median value of this covariate in the database was used for interpolation; if the missing values of a covariate was > 40%, the covariate would be discarded and not incorporated into the final models.

### Model development

#### Summary of basic functional relationship of model development

The date composed of all the dose regimens provided the possibility to seek the potential dose-effect and time-effect relationships for each drug. First, we explore the data graphically, followed by the longitudinal profiles of PASI75, PASI90 and PASI100 were fitted using the hierarchical models with the maximum likelihood estimation method. In this study, we defined the sum of placebo response and pure drug efficacy as the efficacy of the drug group. The further explanation was that pure drug efficacy was equal to the efficacy subtracted the corresponding placebo response. The following is the description of the model:


(1) $${\text{E}}_{i,j,t}={\text{E}}_{placebo,i,t}+{\text{E}}_{drug,i,j,t}$$

(2) $${\text{E}}_{drug,i,j,t}=function\left(drug,\;dose,\;regimen,\;time,\;\theta ,\;X_{ij}\right)$$

In formula 1, E*_placebo,i,t_* and E*_drug,i,j,t_* represent the placebo effect of the *i*th trial at *t* time and the drug effect in the *j*th treatment arm of the *i*th trial at *t* time, respectively. E*_i,j,t_* is the sum of E*_placebo,i,t_* and E*_drug,i,j,t_*, which represents the efficacy of biologics in the *j*th treatment arm of the *i*th trial at *t* time. Studying pure drug efficacy (E*_drug,i,j,t_*) can reduce the bias caused by the placebo effect. A logit translation was implemented to restrict the treatment effect of three efficacy outcomes, which measured as probabilities, to a range of 0–1. E*_drug,i,j,t_* in formula 2 is described as a function that dependent on the following parameters: types, dosages and drug regimens of biological agents, time, fixed-effect model parameters *θ*, trial covariates *X*.

#### Summary of time-varying functional relationship of model development

To begin with, it was assumed that the effects of the drug would not change over time and the achievement of convergence was regarded as the symbol of successful development of basic model. Then, in the course of model development, a time variable, which can describe the time-varying drug effects, was added to create a non-linear model to improve the degree of model fitting. The functional relationship of formula was shown as follows:


(3) $${\text{E}}_{drug}={\text{E}}_{\text{max}drug}\cdot \left(1-{\text{e}}^{-k\cdot time}\right)$$

In formula 3, E_max_ *_drug_* and parameter *k* stand for maximum response for each drug and the rate constant which could describe onset of drug effect, respectively (11, 12).

#### Summary of dosage-varying functional relationship of model development

In the initial stage of the model development, no matter what the dosage of each agent is, we assume their maximum efficacy was a constant which described as a scaling factor, E_max_. Then in the process of exploring to improve the degree of model fitting, different dose–response functional relationships, which came from dividing E_max_ into multiple parameters to match different dose regimens, were implemented to the model. For drugs with multiple options of dosage, a dose–response relationship was estimated by the E_max_ model (formula 4) or log-linear model (formula 5).


(4) $$f=\frac{\text{E max}drug\cdot \text{Dose}}{ED50\;+\;Dose}$$

(5) $$f=\left({\text{E}}_{\text{max}drug}+\beta \cdot log\left(\text{Dose}-\text{constant}\right)\right)$$

For drugs with single option of dosage, the dose–response relationship could be estimated by either simple fixed-effect (**Supplementary Formula 4**) or linear dose–response model (**Supplementary Formula 5**), because it was too tough to handle it in the E_max_ or log-linear model. More detailed instructions about model development are available in the **Supplementary Materials** (Model Development section).

### Model weight determination method

For PASI75, PASI90, PASI100, the determination of the weight was based on the standard error of the observed values and was generated by the following equation (formula 6) with *P* and *N*, avoiding the possible deviations in the final model caused by the extreme outcome values, and ensuring that trials with a larger sample size had a greater weight.


(6) $$Weight=\sqrt{\frac{P\cdot \left(1-P\right)}{N}}$$

### Covariate

After screening according to specific inclusion criteria of covariates, a total of six baseline characteristics were taken into the model as covariates, including percentage of males, age (year), weight (kg), disease duration (year), percentage of patients previously treated with a biological therapy and baseline PASI. The following equation (In formula 7) was applied to determine the possible impact of covariates on therapeutic efficacy. In this function, the *θ* was regarded as the parameter to quantify the covariate characteristic effects.


(7) $${\text{Effect}}_{\text{Covariate}}=\frac{\text{Covariate}\theta }{\text{mean}\left(\text{Covariate}\right)}$$

The development and iteration of the model were based on the analytic dataset and the achievement of convergence was regarded as the symbol of successful development. How to select model depended on the log likelihood ratio and Akaike information criterion at an acceptance *p*-value of 0.05.

### Model evaluation and typical efficacy analysis

After model establishment, the model-fitted time-varying plots and diagnostic plots were used to evaluate the model fitting degree of each trial. A total of 10,000 model parameters were sampled from the final model, which were used to conduct model simulation of drug response at hypothetical time points. (In this study, 12 Week, which was normally as the end of induction treatment was used as the hypothetical time).

### Ranking of adverse events in Bayesian framework approach

The ranking of AEs derived from the data of maximum dose regimens of each drug. For AEs outcomes, they usually reported at the end of the induction phase. In this study, according to an NMA based on random models with the Bayesian method, we compared direct and indirect AEs of biologics with those of placebo. In addition, random models with the Bayesian method also had a use for the comparison between direct and indirect efficacy outcome (PASI75 at the end of induction phase) in biologic group and those in placebo group. Then, we combined the relative ranking (odds ratio compared with placebo) of efficacy and safety outcomes into the one figure.

R software version 4.2.1 [R Core Team (2022)] and the “gnls” function in the “nlme” package version 3.1.159 were operated within the whole process of data exploration as well as model development, evaluation, and simulation. Three packages including “gemtc 1.0-1”, “netmeta 2.5-0” and “ggplot2 3.3.6” were used to estimate the relative ranking of AEs in Bayesian framework approach. Literature quality was assessed by the Review Manager (RevMan), version 5.3.5, The Cochrane Collaboration, 2014.

## Results

### Characteristics of included studies

**Figure 1:**
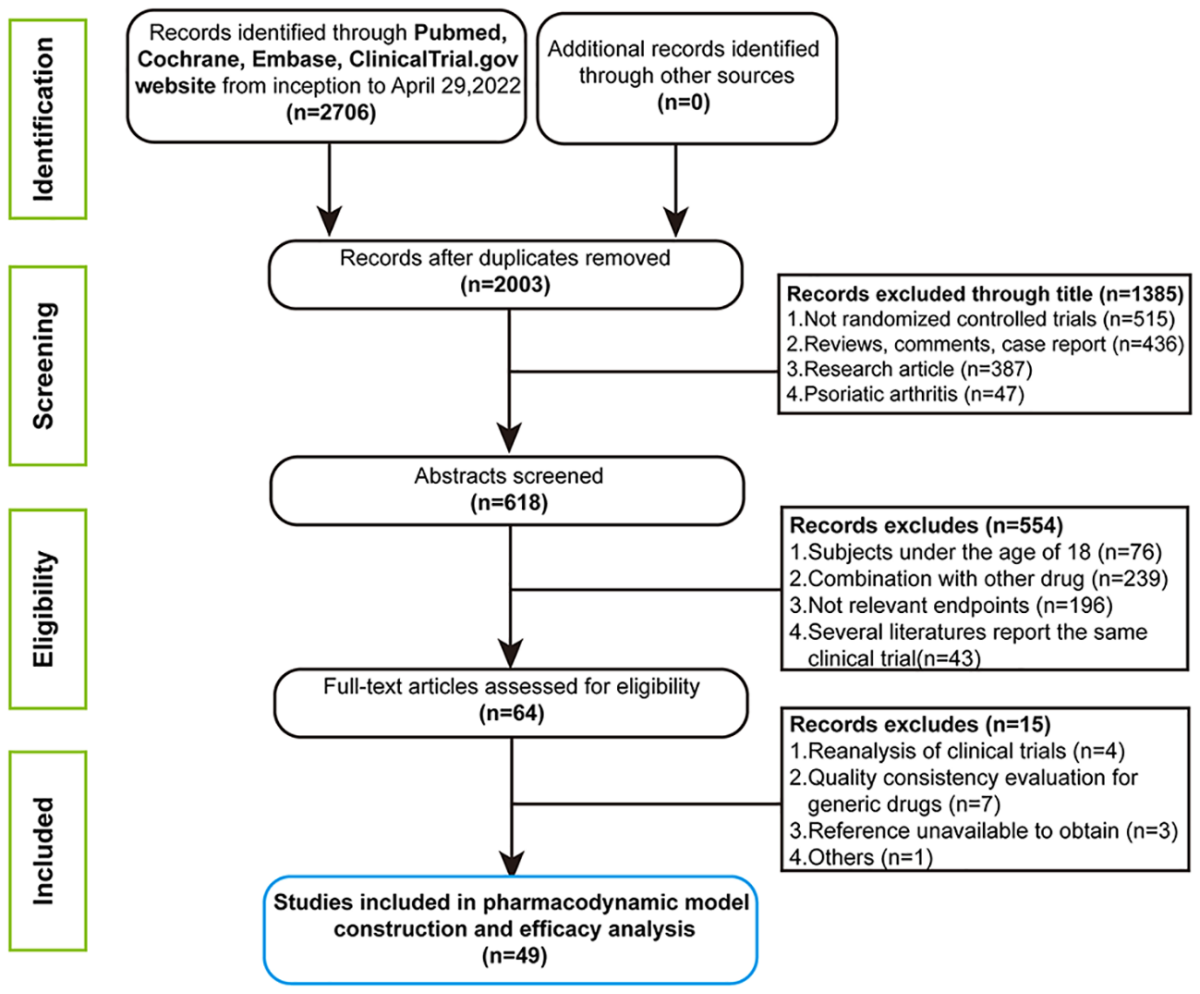
Flow diagram for study selection.

In the initial retrieval phase, 2,706 medical literatures were obtained. Finally, a total of 49 studies including 54 trials, containing 180 treatment arms with 27,808 patients were included for analysis. The flow chart of the complete process screening the included articles is shown in **Figure 1**. The drugs involved in the analysis contained seventeen biological agents. On the basis of the types of drug targets, it could be divided into the six categories: IL-17 or 17 (RA) inhibitor, IL-12/23 inhibitor, IL-17A/17F inhibitor, TNF-a inhibitors, IL-23 inhibitor and CD6 antagonist. The drug classifications and overviews of 54 trials as well as baseline characteristics prescribed in advance are summarized in **Table 1**.

**Table 1:** Summary of available information for each drug in the analysis.

Drug	Trials	Patients	Arms	Route (regimen)	Percentage of males (%)	Age (year)	Weight(kg)	Disease duration (years)	Percentage of patients previously treated with a biological therapy (%)	Baseline PASI
IL- 17A (RA) inhibitor										
Brodalumab	5	3,069	10	s.c. (70, 140, 210mg q2w)	72.72	45.10	83.93	16.52	24.44	22.80
Ixekizumab	4	2,449	10	s.c. (10, 25, 75, 150mg 0,2,4,8w; 160mg 0w followed by 80mg, q2w or q4w)	63.32	45.90	92.20	17.90	28.74	19.37
Secukinumab	10	2,292	22	s.c. (25mg, once or q4w; 75, 150mg q4w; 2×150, 300mg 0,1,2,3,4,8w)	71.64	44.60	88.69	17.87	24.84	21.60
Vunakizumab	1	150	4	s.c. (40, 80, 160, 240mg q4w)	74.67	41.38	88.56	13.48	24.80	22.75
IL-12/23 inhibitor										
Briakinumab	2	277	2	s.c. (200mg 0,4w_100mg 8w)	65.70	44.25	94.65	16.20	10.85	18.90
Ustekinumab	7	2,491	10	s.c. (45, 90mg 0,4w)	72.07	44.90	85.49	17.88	29.22	22.51
IL-17A/17F inhibitor										
Bimekizumab	3	878	7	s.c. (64, 160, 320, 480mg q4w; 320mg 0w followed by 160mg, q4w)	67.81	44.19	88.18	15.83	28.40	19.76
Sonelokimab	1	208	4	s.c. (30, 60, 120mg 0,2,4,8w; 120mg q2w)	72.53	45.60	91.38	18.36	16.80	20.68
TNF-α inhibitor										
Adalimumab	6	1,900	8	s.c. (40, 80mg eow; 80mg 0w followed by 40mg eow)	75.50	44.85	79.98	15.83	26.55	24.43
Certolizumab	4	793	8	s.c. (400mg 0, 2, 4w followed by 200mg q2w; 400mg q2w)	67.33	46.76	86.83	16.95	33.35	21.35
Etanercept	9	2,273	10	s.c. (25, 50mg biw)	67.39	45.22	90.65	18.26	18.92	19.31
Infliximab	3	509	4	i.v. (3mg/kg, 5mg/kg 0, 2, 6w)	69.08	44.63	83.55	16.83	28.80	23.70
IL-23 inhibitor										
Guselkumab	4	1,161	9	s.c. (5, 15, 50, 100, 200mg q4w)	71.51	45.06	85.87	17.55	31.69	22.31
Mirikizumab	1	153	3	s.c. (30, 100, 300mg 0, 8w)	72.00	47.57	88.53	19.03	41.00	19.90
Risankizumab	4	1,118	5	s.c. (75, 150mg 0, 4w)	76.50	49.78	83.14	16.95	34.70	22.30
Tildrakizumab	3	1,543	8	s.c. (5, 25, 100, 200mg 0, 4w)	73.00	45.09	88.67	16.95	18.00	20.85
CD6 antagonist										
Itolizumab	1	180	2	i.v. (0.4mg/kg 0, 1, 2, 3, 4w then 1.6mg/kg q2w; 1.6mg/kg q2w)	79.45	41.60	88.56	16.95	24.80	20.9
Placebo	54	6,364	54		70.60	45.23	86.61	17.12	27.00	21.31
Total	54	27,808	180		71.27	45.10	87.53	17.03	26.27	21.37

RA, receptor A; s.c., subcutaneous injection; i.v., intravenous injection; w, week; eow, every other week; biw, twice weekly; q2w, once every 2 weeks; q4w, once every 4 weeks.

PASI75 endpoint was reported in all 54 selected trials, and PASI90 and PASI 100 were reported in 53 and 39 selected trials, respectively. Detailed information about time points of every outcome reported in included studies and references are displayed in **Supplementary Table S1**. The risk of bias assessments of the included studies are displayed in **Supplementary Figure S1**. The detailed literature quality information of included studies are shown in **Supplementary Figure S2**.

### Final models and typical drug efficacies

#### The time course relationship part in final models

The drug effect measured by PASI75, PASI90 and PASI100 were described by the following equations: For IL-12/23 inhibitors (briakinumab and ustekinumab),


(8) $${\text{E}}_{drug(\text{IL}-12/23)}={\text{E}}_{\text{max}drug(\text{IL}-12/23)}\cdot \left(1-{\text{e}}^{-k\cdot time}\right)$$

E*_drug_*_(_ *_IL-12/23)_* is an exponential function dependent on time, in which *k*-value represents the rate constant describing the onset of IL-12/23 inhibitor drugs and E_max_ *_drug_*_(_ *_IL-12/23)_* stands for maximal efficacy.

For other 5 classes of biologics including IL-17 or 17 (RA) inhibitors, IL-17A/17F inhibitors, TNF-α inhibitors, IL-23 inhibitors and CD6 antagonists,


(9) $${\text{E}}_{drug}=f\left({\text{E}}_{\text{max}drug},dose,\;regimen\right)$$

E*_drug_* is a function dependent on E_max_ *_drug_* (which were estimated individually for each biologic), dose, regimen but independent of time. According to the exponential algorithm (formula 8), the time to reach 50% of the maximum effect (ET_50_) of IL-12/23 inhibitors was evaluated to be about 2.4 weeks (PASI75), 2.5 weeks (PASI90) and 2.7 weeks (PASI100), respectively. In a word, except for the IL-12/23 inhibitor regimen in which a time-varying drug effect was observed by researchers, the other biologics immediately achieved the maximum therapeutic effect.

#### The dose-response relationship part in final models

For the three longitudinal models constructed in this study, the dose-response relationships were not significant for briakinumab. In the PASI75 longitudinal model, the dose-response relationships of certolizumab, sonelokimab and risankizumab were better fit with a log-linear model, while the remaning 13 biologics, the dose-response relationships were better fit with the E_max_ model. In the PASI90 longitudinal model, all biologics exhibited the same dose-response relationships as PASI75 except itolizumab (The RCT study about itolizumab did not report PASI90). In addition, the E_max_ model dose-response relationships were found in 9 biologics and the log-linear model dose-response relationships were found in risankizumab and sonelokimab for the PASI100 longitudinal model. The estimate of the key parameters in the final models of PASI75, PASI90 and PASI100 are provided in **Table 2**.

**Table 2:** Estimate of key parameters in PASI75, PASI90 and PASI100 final models.

Essential factor	Parameter	PASI75		PASI90		PASI100	
		Estimate	95%CI	Estimate	95%CI	Estimate	95%CI
Adalimumab^a^	E_max_	6.42	(3.83, 9.01)	6.30	(3.74, 8.86)	/	/
	ED_50_	38.61	(-1.16, 78.38)	33.60	(-3.18, 75.72)	/	/
Brodalumab^a^	E_max_	7.75	(7.06, 8.44)	7.14	(6.61, 7.67)	6.70	(6.08, 7.32)
	ED_50_	147.70	(117.61, 177.79)	103.92	(81.40, 185.32)	76.19	(55.38, 97.00)
Bimekizumab^a^	E_max_	6.10	(5.67, 6.53)	5.96	(5.53, 6.39)	5.55	(5.00, 6.10)
	ED_50_	35.69	(16.31, 55.07)	25.75	(9.88, 35.63)	24.22	(6.21, 42.23)
Etanercept^a^	E_max_	3.74	(2.88, 4.60)	4.21	(2.22, 6.20)	/	/
	ED_50_	15.40	(1.19, 29.61)	18.09	(-13.50, 49.68)	/	/
Guselkumab^a^	E_max_	4.52	(4.27, 4.77)	4.66	(4.25, 5.07)	3.99	(3.45, 4.53)
	ED_50_	2.47	(1.28, 3.66)	2.46	(0.93, 3.39)	2.97	(0.31, 5.63)
Ixekizumab^a^	E_max_	5.41	(5.16, 5.66)	5.64	(5.25, 6.03)	5.34	(4.70, 5.98)
	ED_50_	5.50	(3.68, 7.32)	3.22	(1.53, 4.91)	4.35	(1.79, 6.91)
Itolizumab^a^	E_max_	3.48	(2.05, 4.91)	/	/	/	/
	ED_50_	12.42	(-0.46, 25.30)	/	/	/	/
Infliximab^a^	E_max_	6.17	(4.62, 7.72)	5.02	(2.72, 7.32)	/	/
	ED_50_	130.76	(18.91, 242.61)	93.81	(-98.95, 286.57)	/	/
Mirikizumab^a^	E_max_	4.21	(3.15, 5.27)	3.96	(2.73, 5.19)	5.45	(1.16, 9.74)
	ED_50_	7.26	(1.14, 13.38)	13.24	(1.56, 24.92)	8.41	(-4.56, 21.38)
Secukinumab^a^	E_max_	5.92	(5.51, 6.33)	6.15	(5.62, 6.68)	5.90	(5.06, 6.74)
	ED_50_	71.14	(53.91, 88.37)	75.31	(53.93, 96.69)	82.16	(42.28, 122.08)
Tildrakizumab^a^	E_max_	3.42	(3.17, 3.67)	4.13	(3.60, 4.66)	3.81	(2.97, 4.65)
	ED_50_	5.17	(2.35, 7.99)	6.98	(0.20, 13.76)	5.38	(-3.02, 13.78)
Ustekinumab^a^	E_max_	4.38	(3.91, 4.85)	4.23	(3.68, 4.78)	3.91	(3.03, 4.79)
	ED_50_	8.18	(2.91, 13.45)	5.07	(-0.71, 10.85)	-0.21	(-8.77, 8.35)
Vunakizumab^a^	E_max_	5.71	(4.79, 6.63)	4.70	(3.58, 5.82)	4.76	(2.86, 6.66)
	ED_50_	44.98	(22.81, 67.15)	34.06	(9.82, 58.30)	35.78	(-0.34, 71.90)
Certolizumab^b^	E_max_	2.69	(1.26, 4.12)	3.21	(1.54, 4.88)	/	/
	β	0.12	(-0.13, 0.37)	4.5×10^-2^	(-0.23, 0.32)	/	/
Risankizumab^b^	E_max_	3.92	(-3.66-11.50)	3.42	(0.62, 6.22)	2.46	(-0.65, 5.57)
	β	0.12	(-1.44, 1.68)	0.23	(-0.33, 0.79)	0.46	(-0.16, 1.08)
Sonelokimab^b^	E_max_	2.30	(0.54, 4.06)	2.78	(1.67, 3.89)	3.49	(2.04, 4.94)
	β	0.60	(0.17, 1.03)	0.59	(0.36, 0.52)	0.36	(0.07, 0.65)
Rate constant	*K_IL-12/23_*	0.29	(0.24, 0.36)	0.28	(0.22, 0.36)	0.26	(0.18, 0.38)
Covariates	Age (year)	/	/	0.20	(-0.19, 0.59)	0.41	(-0.13, 0.95)
	Body weight (kg)	/	/	-0.83	(-1.18, -0.48)	-0.51	(-1.07, 0.05)
	Disease duration (year)	/	/	/	/	-0.26	(-0.47, -0.05)
	Prior biological therapy (%)	5.36×10^-2^	(1.49×10^-2^, 9.23×10^-2^)	5.50×10^-2^	(1.60×10^-2^, 9.40×10^-2^)	/	/

PASI, Psoriasis Area and Severity Index score; PASI75, PASI90 and PASI100, the proportion of patients achieving ≥75%, 90% and 100% respectively reduction from baseline PASI score; ^a^ E_max_ model with a E_max_ and a ED_50_ was used for the dose-response relationship; ^b^ log-linear model with a E_max_ and a β was used for the dose-response relationship.

#### Covariates in final models

Correlation between six covariates (percentage of males, age (year), body weight (kg), disease duration (year), percentage of patients previously treated with a biological therapy and baseline PASI) and drug efficacy was tested. For PASI75 model, the estimated covariate parameters of positive value (5.36×10^-2^, [95%CI: 1.49×10^-2^ to 9.23×10^-2^]) for percentage of patients previously treated with a biological therapy indicated that the patients with higher percentage of patients previously treated with a biological therapy were anticipated to get better curative effect. The parameters for age (0.20 [95%CI: -0.19 to 0.59]), body weight (−0.83 [95%CI: -1.18 to -0.48]) and percentage of patients previously treated with a biological therapy (5.50×10^-2^ [95%CI: 1.60×10^-2^ to 9.40×10^-2^]) in the PASI90 model meant that patients with older age, lower body weight and higher percentage of patients previously treated with a biological therapy were assumed to get greater efficacy in PASI90. However, for PASI100 model, the estimated covariate parameters of negative value (-0.51, [95%CI: -1.07 to 0.05]) and (-0.26, [95%CI: -0.47 to -0.05]) for body weight and disease duration suggested that the patients with lower of those factors were anticipated to get better curative effect. Detailed information is given in **Table 2**.

#### The fitted time-course plots of final models

**Figure 2:**
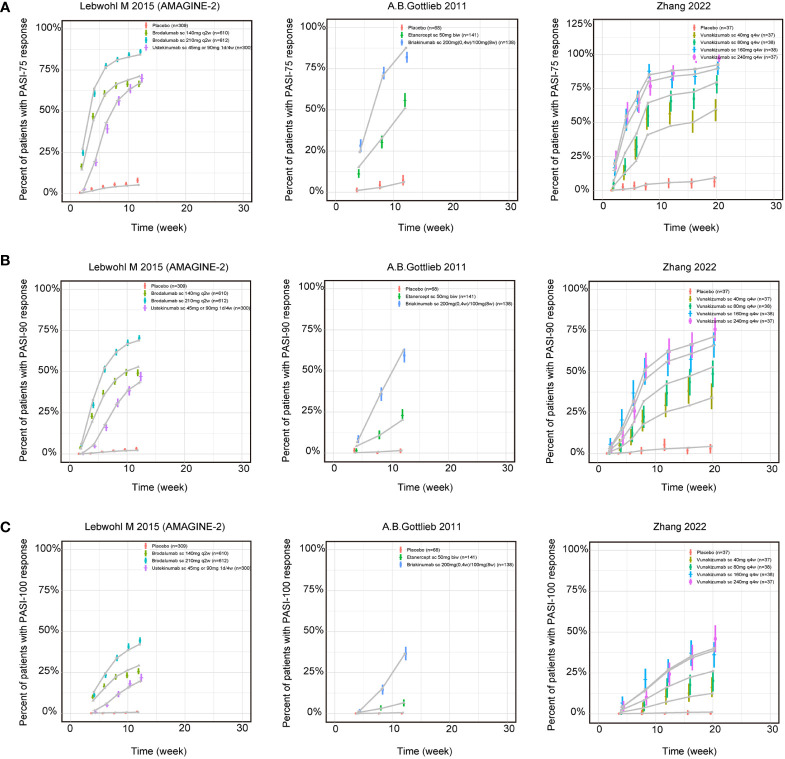
Model fitted time-course plots of response rate for **(A)** PASI75, **(B)** PASI90 and **(C)** PASI100 for representative trials. Color symbols and vertical bars are observed mean and calculated weight of time points; gray symbols and lines are the model predictions. PASI, Psoriasis Area and Severity Index score; PASI75, PASI90 and PASI100, the proportion of patients achieving ≥75%, 90% and 100% respectively reduction from baseline PASI; s.c., subcutaneous injection; w, week; biw, twice weekly; q2w, once every 2 weeks; q4w, once every 4 weeks.

The fitted time-course plots of representative studies for three longitudinal models are displayed in **Figure 2**. The fitted time-course plots of the other studies can be obtained in **Supplementary Materials** (**Figure S3-S5**).

#### Model simulation and typical drug efficacies

In this study, four covariates were included in the final model. Hence, a typical trial which could indicate the common feature of included trials was assumed for the simulation with a hypothetical population (average value: 45 years old, weight 88.56 kg, 16.96 years of disease duration and 24.8% had prior biological therapy). To account for the placebo effect and to evaluate the relative clinical efficacy, a longitudinal placebo effect model was established for three outcomes.

**Figure 3:**
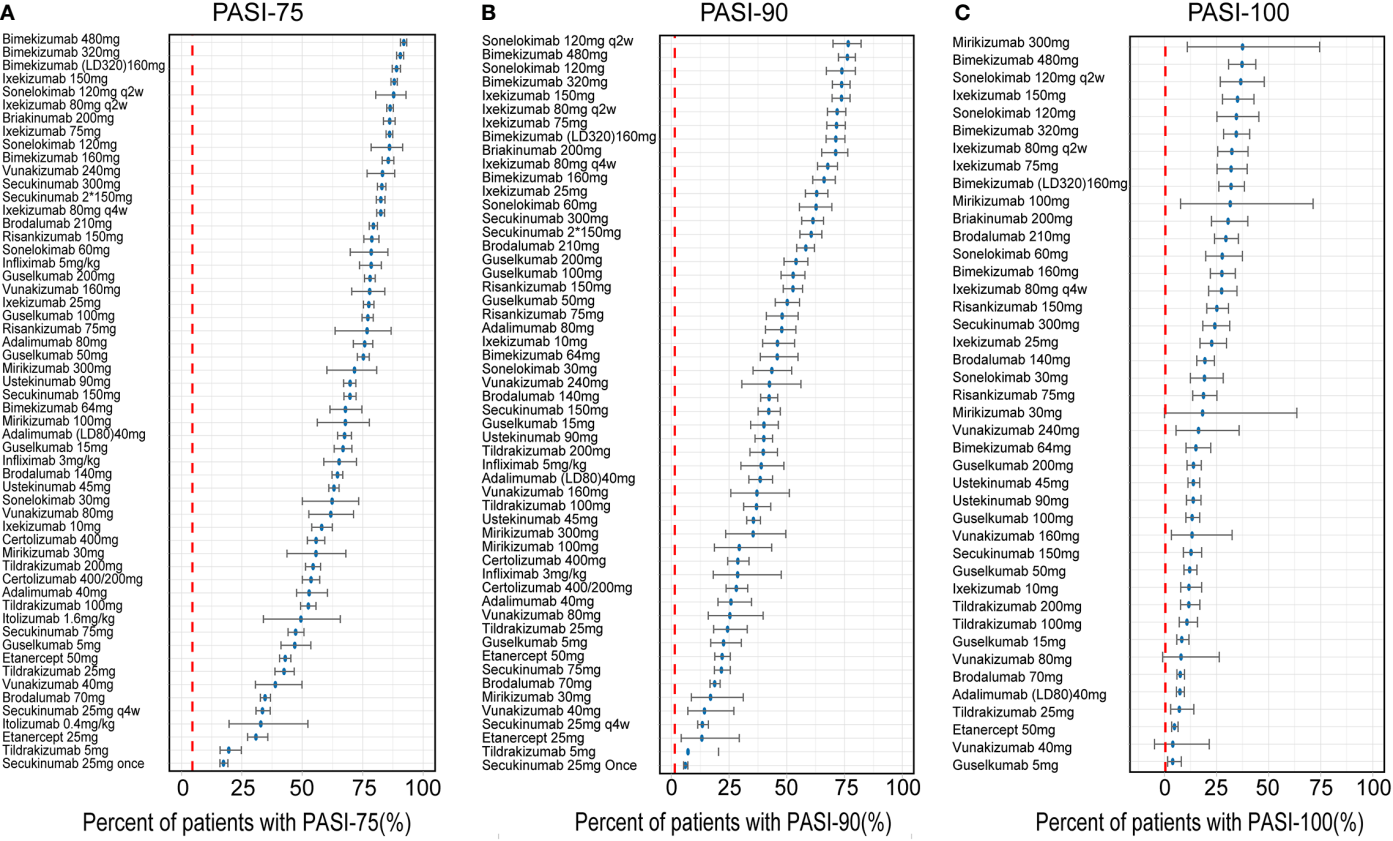
Ranking of the treatments by predicting placebo-corrected median percent of patients with **(A)** PASI75, **(B)** PASI90, and **(C)** PASI100 at week 12 (descending order). Point estimates and 95% CIs were predicted from a model simulation of N = 10,000. Red dashed lines represent simulated placebo efficacy. PASI, Psoriasis Area and Severity Index score; PASI75, PASI90 and PASI100, the proportion of patients achieving ≥75%, 90% and 100% respectively reduction from baseline PASI; LD, loading dose.

How are the placebo-corrected median of treatment effect ranked at the time point of 12 weeks for three longitudinal models is shown in the **Figure 3**. The **Figure 3A** shows the model simulation and typical biologic efficacies of the PASI75, where the placebo effect is estimated to be 4.45%. The results showed that bimekizumab 480mg had the best typical drug efficacy in PASI75 model, in which has a narrow 95% credible interval (95% CI) (median: 92.03%, 95% CI: 90.65% to 93.23%), with bimekizumab 320mg (median: 90.57%, 95% CI: 88.96% to 91.93%) and bimekizumab (LD320mg)160mg (median: 89.01%, 95% CI: 87.21% to 90.59%) following. For PASI90 model (**Figure 3B**), with a placebo effect estimated as 1.48%, sonelokimab 120 mg augmented load (q2w) was also calculated to have the best typical drug efficacy as 76.60% (95% CI:69.98% to 82.14%), followed by bimekizumab 480mg (median: 76.25%, 95%CI: 72.34% to 79.66%) and sonelokimab 120 mg normal load (median: 73.85%, 95% CI:67.09% to 79.65%). For the model simulation and typical biologic efficacies of PASI100 (**Figure 3C**), where the placebo effect was estimated to be 0.41%, mirikizumab 300mg was predicted to have the highest efficacy as 38.33% in PASI100 but with a large 95% CI (11.25% to 74.75%). Bimekizumab 480mg (median: 37.30%, 95% CI: 30.82% to 43.90%) and sonelokimab 120 mg q2w (median: 36.94%, 95% CI: 26.67% to 48.25%) were predicted to have the second and third highest drug efficacy.

From the categories of biological agents, IL-17A/17F inhibitor (bimekizumab, sonelokimab), IL-17A (RA) inhibitor (ixekizumab, vunakizumab) and IL-12/23 inhibitor (briakinumab) were predicted to have the highest efficacy in PASI75, ranking in the top five drugs. But in PASI90, the ordering was slightly different from PASI75, IL-17A/17F inhibitor (sonelokimab, bimekizumab), IL-17A (RA) inhibitor (ixekizumab, secukinumab) and IL-12/23 inhibitor (briakinumab) rounded out the top five. In addition, compared with PASI90, mirikizumab replaced secukinumab and moved into the top five in PASI100.

### The incidence of AEs in RCTs (vs placebo at the end of induction period)

As far as the incidence of AEs, the maximum dose regimens arms of 17 biologics were distributed in 34 articles, in which a total of 40 trials, containing 80 treatment arms with 13,583 patients were included for analysis. The network plot presenting 40 trials data contributing evidence comparing incidence rate of AEs are shown in **Supplementary Figure S7**.

**Figure 4:**
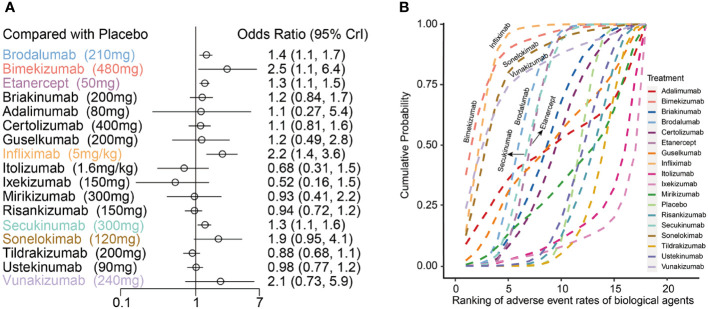
**(A)** Forest plots of network meta-analysis results for adverse events with placebo as reference; **(B)** Cumulative probability diagram of adverse events of 17 biological agents.

Compared to placebo, bimekizumab 480mg (OR:2.50, 95%CI:1.10 to 6.40), infliximab 5mg/kg (OR:2.20, 95%CI: 1.4 0to 3.60), brodalumab 210mg (OR: 1.40, 95%CI: 1.10 to 1.70), etanercept 50mg (OR: 1.30, 95%CI: 1.10 to 1.50) and secukinumab 300mg (OR: 1.30, 95%CI: 1.10 to 1.60) significantly increased the incidence of AEs. The relative incidence rate of AEs among 17 biologics compared with placebo can be seen in **Figure 4A**. The results of incidence rate of AEs in **Figure 4B** were based on the surface under the cumulative ranking curve (SUCRA), which showed that infliximab 5mg/kg had the highest probability to rank as the worst treatment and presented the highest SUCRA value (88.65%), followed by bimekizumab 480mg (88.55%), sonelokimab 120mg (81.64%) and vunakizumab 240mg (78.02%). In addition, brodalumab 210mg, secukinumab 300mg and etanercept 50mg also showed higher SUCRA values of 66.36%, 61.93% and 60.67%, respectively.

**Figure 5:**
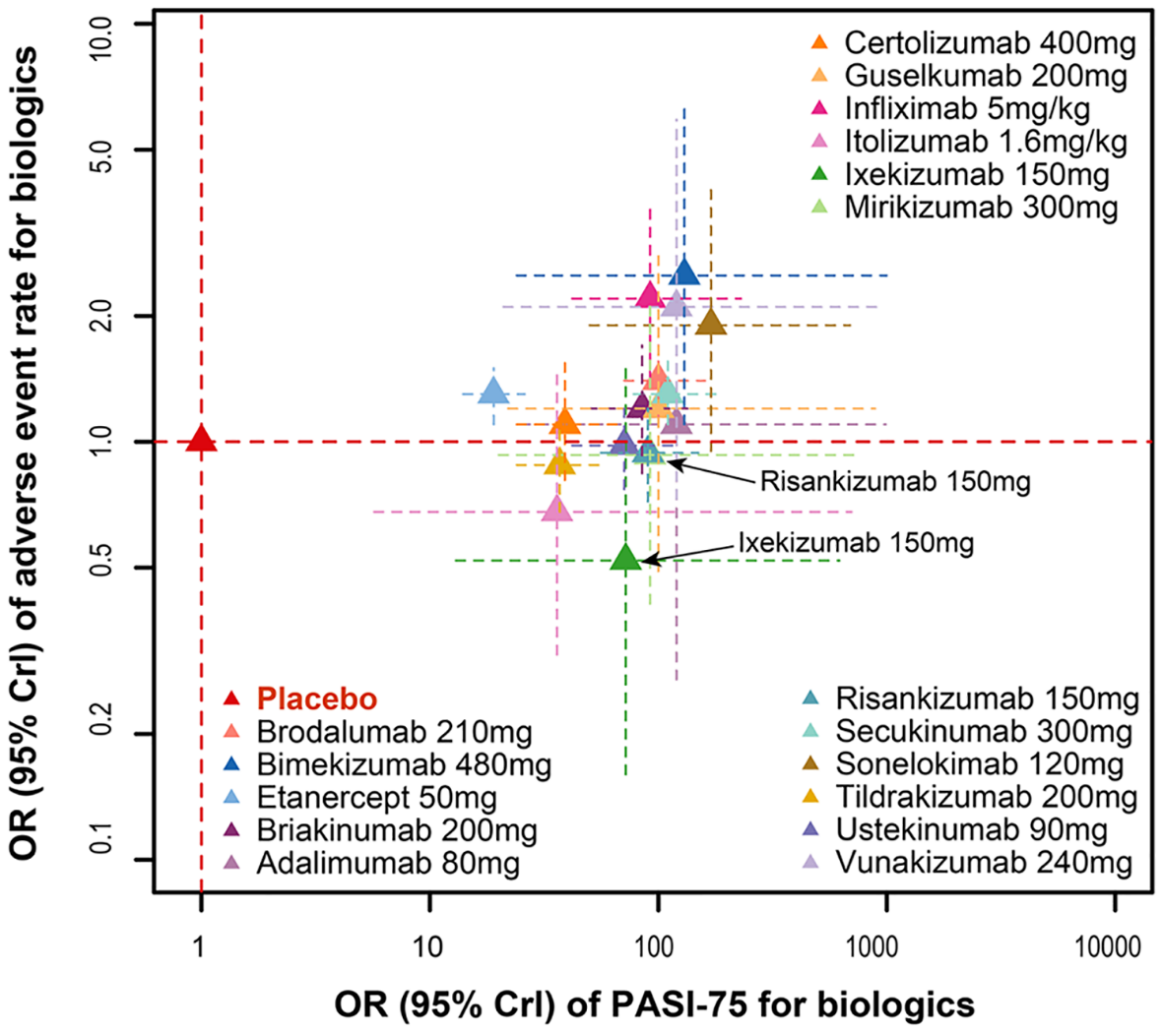
Ranking of biological drugs according to efficacy and safety at the end of induction period. Red dashed lines represent placebo effects. OR, odd ratio.

The odds ratios for PASI75 and AEs of 17 biologics compared with placebo at the end of the induction phase are shown in **Figure 5**. The results showed that infliximab 5mg/kg ([PASI75, OR: 93.00, 95%CI: 42.00 to 230.00]; [AE, OR: 2.20, 95%CI: 1.40 to 3.60]) and bimekizumab 480mg ([PASI75, OR: 130.00, 95%CI: 28.00 to 1200.00]; [AE, OR: 2.50, 95%CI: 1.10 to 6.40]) had the significantly better efficacy, but also accompanied by the obviously higher incidence of AEs. However, there are still some biologics with markedly good efficacy and the trend of decreasing incidence of AEs, such as risankizumab 150 mg ([PASI75, OR: 89.00, 95%CI: 56.00 to 150.00]; [AEs, OR: 0.94, 95%CI: 0.72 to 1.20]). Furthermore, ixekizumab 150mg ([PASI75, OR: 70.00, 95%CI: 12.00 to 690.00]; [AEs, OR: 0.52, 95%CI: 0.16 to 1.50]) had significantly better efficacy with a higher incidence of AEs, had a significantly efficacy in PASI75.

## Discussion

Our research applied a quantitative method to estimate the comparative efficacy of biologic therapies on MSPP. Moreover, the method could quantify not only the efficacy of each drug, but also the effects of time, dosage and covariates. The **Figure 3** demonstrated that bimekizumab administered as three dosage regimens (480mg, 320mg q4w and 320mg 0w followed by 160mg q4w) all provided the favorable efficacy response of PASI75, PASI90 and PASI100 at week 12. These results were known from trials (13–15) corresponded to the NMA conducted by Armstrong et al. (16) as well as published recently, in which the results showed that compared to other biologic and non-biologic treatments at 10 to 16 weeks, bimekizumab 320mg had obvious advantage in achievement of higher PASI response rate. These results were known from trials (11–13) and corresponded to the NMA conducted by Armstrong et al. (14) as well as published recently, in which the results showed that compared to other biologic and non-biologic treatments at 10 to 16 weeks, bimekizumab 320mg had obvious advantage in achievement of higher PASI response rate. In addition, from the variety of biological agents, sonelokimab, bimekizumab and ixekizumab were predicted to have the highest efficacy in all three longitudinal models, ranking in the top five. These findings suggested that the efficacy trend is similar across all PASI response levels: IL-17A/17F inhibitor and IL-17A (RA) inhibitor treatments were the most effective treatments among the seventeen biological agents. The IL-17A, which originated from activated T cells, nowadays is considered as a crucial pro-inflammatory cytokine in chronic immune-mediated inflammatory diseases such as MSPP (17). Among IL-17 family members, IL-17A and IL-17F sharing almost 50% structural homology, are closest in sequence (18). The current concept is that IL-17A and IL-17F are both upregulated in MSPP (19, 20). Just as the therapeutic efficacy of secukinumab and ixekizumab bore out in MSPP (21, 22), it is an effective clinical strategy to inhibit downstream of IL-17A cytokines directly. However, given the enrichment of IL-17F in MSPP (23), it has been demonstrated that compared to inhibiting IL-17A only, if both IL-17A and IL-17F cytokines are inhibited selectively at the same time, there will be a greater clinical benefit (24). In a word, bimekizumab and ixekizumab were the most effective treatments. Sonelokimab (also known as M1095), which is ranked as the most effective treatments among the biologics in this study, is a novel trivalent nanobody. Limited by the sample size and the number of RCT, the efficacy of sonelokimab needs to be verified by more studies in the future.

Our study provided not only a more detailed ranking of efficacy, but also a new insight into the time-course, dose–response relationships of biologics by applying longitudinal models compared to predecessor’s NMA. Firstly, the data of efficacy corresponding to different time points were put into an exponential model to describe the underlying time-varying drug effect. However, a time-varying drug effect was only found in IL-12/23 inhibitor, this may indicate that the efficacy of briakinumab and ustekinumab (belongs to IL-12/23 inhibitor) increased with time, and the other biologics immediately achieved the maximum therapeutic effect. Secondly, different dose–response functions, which came from dividing E_max_ into multiple parameters to match different dose regimens, were used to describe the impact of different dose-response relationships on the treatment efficacy. The results suggest that apart from the briakinumab in which due to single option of dosage, the dose-varying efficacy model had not been successfully established, E_max_ or log linear model had been established successfully for other drugs, which demonstrating that the efficacy of the drug increases with increasing dosage. Those are of great importance for physicians to make appropriate treatment strategies. For example, through established longitudinal models we simulated the efficacy of sonelokimab across four dosage regimens (30mg, 60mg, 120mg normal load and 120mg augmented load) at the end of induction period. This provides a comprehensive quantitative comparison of differences and similarities across 17 biologics. Thus, our results will enable physicians and patients shared decision-making as suggested by the American Academy of Dermatology (AAD) and National Psoriasis Foundation (NPF) that “The choice of systemic psoriasis therapy is individualized for each patient and involves the consideration of patients’ disease severity, comorbidities, lifestyle, and patient preferences (25–27).”

The advantage of MBMA is that it could quantify the influence of covariates on the therapeutic efficacy (28). In this study, the patients with lower body weight were proved to get more promotion in PASI90 and PASI100, which was consistent with previous studies (29). Our analysis indicated that older patients were also revealed to achieve significant efficacy in PASI90 and PASI100. However, a previous observational study (30) suggested that biological agents for psoriasis showed similar efficacy no matter in the old or in the young. The reason why the differences emerged is that our identification of covariates was based on the comparative drug efficacy, instead of based on the absolute treatment effects of small sample size retrospective like other studies. In the PASI100 longitudinal model, patients with shorter disease duration exhibited better improvement. These consistent with the study of MPSS natural history which suggested that the longer duration of the disease is, the more severe and complicated conditions are (2), so the correlation between the duration and severity of the disease may be positive. In addition, our study indicated that the patient previously treated with a biological therapy was considered to show more benefit in the PASI75 and PASI90 models.

In terms of the incidence of AEs, bimekizumab 480mg, infliximab 5mg/kg, brodalumab 210mg, etanercept 50mg and secukinumab 300mg were significantly higher than the placebo (**Figure 4A**). At the same time, according to the SUCRA results for AEs in **Figure 4B**, infliximab 5mg/kg and bimekizumab 480mg had the highest probability to rank as the worst treatment. These results indicated that the most effective treatment often come with significant side effects. The biologics which could achieve a balance between those efficacy and safety are displayed in the lower middle position corner of **Figure 5**, so that ixekizumab and risankizumab may be the better choices for the treatment of MSPP. This conclusion is consistent with the results of previous literatures (8, 31), which reported that the efficacy as well as safety of ixekizumab and risankizumab showed relative stability and balanced performance. In March 2016, ixekizumab directed against IL-17A, was approved for the treatment of MSPP. The safety information of drug instructions of ixekizumab is reassuring, but there still have some adverse side effects, such as nasopharyngitis, upper respiratory infections, headache, injection sites reactions, diarrhea and mild neutropenia, were observed in phase II and III clinical trials commonly (32–34). In addition, the neutropenia and mucocutaneous candidiasis caused by ixekizumab are the adverse side effects that doctors and researchers pay close attention to it. However, these AEs, which severity was usually mild to moderate, were rarely observed and hardly lead to discontinuation of the ixekizumab. Nevertheless, laboratory monitoring of neutrophil blood count and clinical monitoring of skin and mucosal infection are necessary during ixekizumab treatment (35). Risankizumab, an IL-23 inhibitor whose target is the p19 subunit of IL-23, were significantly more effective than the inhibitors of TNF-α as shown in the previous studies (31, 36, 37). The upper respiratory tract infection was the most common AEs reported in trials with Risankizumab (38). To sum up, IL-17A inhibitor (ixekizumab) and IL-23 inhibitor (risankizumab, tildrakizumab and guselkumab) performed superior to the others in terms of safety. However, further researches are essential to enrich the information of the safety profile of these agents for the treatment of MSPP in routine practice.

There are still following limitations in our study. Firstly, some of the included literatures do not provide the data of PASI90 and/or PASI100, as a result, the longitudinal models of PASI90 and PASI100 contain less than 17 kinds of biological agents. Secondly, since the target population of this study was MSPP patients, the biologic efficacy prediction model established in this study was limited in its generalizability and may be not available to mild psoriasis patients. Thirdly, the data on the efficacy of some biologics remain inadequate, which could not make sure the absolute accuracy and reliability of the three models, for example, the ED_50_ for ustekinomab in the PASI100 model was estimated to be negative. Thus, more studies should be carried out in the future.

In conclusion, bimekizumab and ixekizumab treatments were the most effective treatments among the biologics. The efficacy of sonelokimab needs to be verified by more studies in the future. As for the covariates, the patients’ age, body weight, disease duration and percentage of patients previously treated with a biological therapy were identified had the impact on drug efficacy. In addition, we found that the efficacy as well as safety of ixekizumab and risankizumab showed relative stability and balanced performance.

## Data availability statement

The original contributions presented in the study are included in the article/**Supplementary Material**. Further inquiries can be directed to the corresponding author.

## Ethics statement

Ethical review and approval was not required for the study on human participants in accordance with the local legislation and institutional requirements. Written informed consent for participation was not required for this study in accordance with the national legislation and the institutional requirements.

## Author contributions

Authors WG, LZ, and RZ were responsible for study conception and design; authors WG, BY, and YY were responsible for acquisition of data; authors WG, BY, LZ, and RZ were responsible for data analysis, and drafting and revision of the manuscript. All authors contributed to the article and approved the submitted version.

## References

[1] Boca AN Ilies RF Saccomanno J Pop R Vesa S Tataru AD. Sea Buckthorn extract in the treatment of psoriasis. Exp Ther Med (2019) 17(2):1020–3. doi: 10.3892/etm.2018.6983 PMC6327666 30679968

[2] Armstrong AW Read C. Pathophysiology, clinical presentation, and treatment of psoriasis: a review. Jama (2020) 323(19):1945–60. doi: 10.1001/jama.2020.4006 32427307

[3] Aira LE López-Requena A Fuentes D Sánchez L Pérez T Urquiza A. Immunological and histological evaluation of clinical samples from psoriasis patients treated with anti-CD6 itolizumab. MAbs (2014) 6(3):783–93. doi: 10.4161/mabs.28376 PMC4011922 24594862

[4] Menter A Korman NJ Elmets CA Feldman SR Gelfand JM Gordon KB. Guidelines of care for the management of psoriasis and psoriatic arthritis: section 6. guidelines of care for the treatment of psoriasis and psoriatic arthritis: case-based presentations and evidence-based conclusions. J Am Acad Dermatol (2011) 65(1):137–74. doi: 10.1016/j.jaad.2010.11.055 21306785

[5] Armstrong AW Puig L Joshi A Skup M Williams D Li J. Comparison of biologics and oral treatments for plaque psoriasis: a meta-analysis. JAMA Dermatol (2020) 156(3):258–69. doi: 10.1001/jamadermatol.2019.4029 PMC7042876 32022825

[6] Chima M Lebwohl M. TNF inhibitors for psoriasis. Semin Cutan Med Surg (2018) 37(3):134–42. doi: 10.12788/j.sder.2018.039 30215629

[7] Jeon C Sekhon S Yan D Afifi L Nakamura M Bhutani T. Monoclonal antibodies inhibiting IL-12, -23, and -17 for the treatment of psoriasis. Hum Vaccin Immunother (2017) 13(10):2247–59. doi: 10.1080/21645515.2017.1356498 PMC5647990 28825875

[8] He H Wu W Zhang Y Zhang M Sun N Zhao L. Model-based meta-analysis in psoriasis: a quantitative comparison of biologics and small targeted molecules. Front Pharmacol (2021) 12:586827. doi: 10.3389/fphar.2021.586827 34276352 PMC8281289

[9] Iznardo H Puig L. Dual inhibition of IL-17A and IL-17F in psoriatic disease. (2021) Ther Adv Chronic Dis 12:20406223211037846. doi: 10.1177/20406223211037846 34408825 PMC8366125

[10] Higgins JP Altman DG Gøtzsche PC Jüni P Moher D Oxman AD. The cochrane collaboration’s tool for assessing risk of bias in randomised trials. Bmj (2011) 343:d5928. doi: 10.1136/bmj.d5928 22008217 PMC3196245

[11] Lin D Shkedy Z Yekutieli D Amaratunga D Bijnens L. Modeling dose-response microarray data in early drug development experiments using r: order-restricted analysis of microarray data. Springer Publishing Company, Incorporated (2012). Available at: https://link.springer.com/book/10.1007/978-3-642-24007-2.

[12] Pérez-Urizar J Granados-Soto V Flores-Murrieta FJ Castañeda-Hernández G. Pharmacokinetic-pharmacodynamic modeling: why? Arch Med Res (2000) 31(6):539–45. doi: 10.1016/s0188-4409(00)00242-3 11257318

[13] Papp KA Merola JF Gottlieb AB Griffiths CEM Cross N Peterson L. Dual neutralization of both interleukin 17A and interleukin 17F with bimekizumab in patients with psoriasis: results from BE ABLE 1, a 12-week randomized, double-blinded, placebo-controlled phase 2b trial. J Am Acad Dermatol (2018) 79(2):277–286.e10. doi: 10.1016/j.jaad.2018.03.037 29609013

[14] Reich K Papp KA Blauvelt A Langley RG Armstrong A Warren RB. Bimekizumab versus ustekinumab for the treatment of moderate to severe plaque psoriasis (BE VIVID): efficacy and safety from a 52-week, multicentre, double-blind, active comparator and placebo controlled phase 3 trial. Lancet (2021) 397(10273):487–98. doi: 10.1016/s0140-6736(21)00125-2 33549193

[15] Gordon KB Foley P Krueger JG Pinter A Reich K Vender R. Bimekizumab efficacy and safety in moderate to severe plaque psoriasis (BE READY): a multicentre, double-blind, placebo-controlled, randomised withdrawal phase 3 trial. Lancet (2021) 397(10273):475–86. doi: 10.1016/s0140-6736(21)00126-4 33549192

[16] Armstrong A Fahrbach K Leonardi C Augustin M Neupane B Kazmierska P. Efficacy of bimekizumab and other biologics in moderate to severe plaque psoriasis: a systematic literature review and a network meta-analysis. Dermatol Ther (Heidelb) (2022) 12(8):1777–92. doi: 10.1007/s13555-022-00760-8 PMC9357587 35798920

[17] Schett G Elewaut D McInnes IB Dayer JM Neurath MF. How cytokine networks fuel inflammation: toward a cytokine-based disease taxonomy. Nat Med (2013) 19(7):822–4. doi: 10.1038/nm.3260 23836224

[18] Hymowitz SG Filvaroff EH Yin JP Lee J Cai L Risser P. IL-17s adopt a cystine knot fold: structure and activity of a novel cytokine, IL-17F, and implications for receptor binding. EMBO J (2001) 20(19):5332–41. doi: 10.1093/emboj/20.19.5332 PMC125646 11574464

[19] Johnston A Fritz Y Dawes SM Diaconu D Al-Attar PM Guzman AM. Keratinocyte overexpression of IL-17C promotes psoriasiform skin inflammation. J Immunol (2013) 190(5):2252–62. doi: 10.4049/jimmunol.1201505 PMC3577967 23359500

[20] Papp KA Weinberg MA Morris A Reich K. IL17A/F nanobody sonelokimab in patients with plaque psoriasis: a multicentre, randomised, placebo-controlled, phase 2b study. Lancet (2021) 397(10284):1564–75. doi: 10.1016/s0140-6736(21)00440-2 33894834

[21] Langley RG Elewski BE Lebwohl M Reich K Griffiths CE Papp K. Secukinumab in plaque psoriasis–results of two phase 3 trials. N Engl J Med (2014) 371(4):326–38. doi: 10.1056/NEJMoa1314258 25007392

[22] Gordon KB Blauvelt A Papp KA Langley RG Luger T Ohtsuki M. Phase 3 trials of ixekizumab in moderate-to-Severe plaque psoriasis. N Engl J Med (2016) 375(4):345–56. doi: 10.1056/NEJMoa1512711 27299809

[23] Kolbinger F Loesche C Valentin MA Jiang X Cheng Y Jarvis P. β-defensin 2 is a responsive biomarker of IL-17A-driven skin pathology in patients with psoriasis. J Allergy Clin Immunol (2017) 139(3):923–932.e8. doi: 10.1016/j.jaci.2016.06.038 27502297

[24] Reich K Warren RB Lebwohl M Gooderham M Strober B Langley RG. Bimekizumab versus secukinumab in plaque psoriasis. N Engl J Med (2021) 385(2):142–52. doi: 10.1056/NEJMoa2102383 33891380

[25] Menter A Strober BE Kaplan DH Kivelevitch D Prater EF Stoff B. Joint AAD-NPF guidelines of care for the management and treatment of psoriasis with biologics. J Am Acad Dermatol (2019) 80(4):1029–72. doi: 10.1016/j.jaad.2018.11.057 30772098

[26] Elmets CA Leonardi CL Davis DMR Gelfand JM Lichten J Mehta NN. Joint AAD-NPF guidelines of care for the management and treatment of psoriasis with awareness and attention to comorbidities. J Am Acad Dermatol (2019) 80(4):1073–113. doi: 10.1016/j.jaad.2018.11.058 30772097

[27] Chat VS Uppal SK. Comparison of guidelines for the use of ustekinumab for psoriasis in the united states. Europe United Kingdom: A Crit appraisal Compr Rev (2021) 34(4):e14974. doi: 10.1111/dth.14974 33991048

[28] Mandema JW Gibbs M Boyd RA Wada DR Pfister M. Model-based meta-analysis for comparative efficacy and safety: application in drug development and beyond. Clin Pharmacol Ther (2011) 90(6):766–9. doi: 10.1038/clpt.2011.242 22089340

[29] Owczarczyk-Saczonek A Placek W. Compounds of psoriasis with obesity and overweight. Postepy Hig Med Dosw (Online) (2017) 71(1):761–72. doi: 10.5604/01.3001.0010.3854 28894050

[30] Momose M Asahina A Hayashi M Yanaba K. Biologic treatments for elderly patients with psoriasis. (2017) J Dermatol 44(9):1020–3. doi: 10.1111/1346-8138.13853 28439956

[31] Xu S Gao X Deng J Yang J Pan F. Comparative efficacy and safety of biologics in moderate to severe plaque psoriasis: a multiple-treatments meta-analysis. J Dtsch Dermatol Ges (2021) 19(1):47–56. doi: 10.1111/ddg.14308 33377312

[32] Hawkes JE Chan TC Krueger JG. Psoriasis pathogenesis and the development of novel targeted immune therapies. J Allergy Clin Immunol (2017) 140(3):645–53. doi: 10.1016/j.jaci.2017.07.004 PMC5600287 28887948

[33] Spuls PI Hooft L. Brodalumab and ixekizumab, anti-interleukin-17-receptor antibodies for psoriasis: a critical appraisal. Br J Dermatol (2012) 167(4):710–3; discussion 714-5. doi: 10.1111/bjd.12025 23013312

[34] Griffiths CE Reich K Lebwohl M van de Kerkhof P Paul C Menter A. Comparison of ixekizumab with etanercept or placebo in moderate-to-severe psoriasis (UNCOVER-2 and UNCOVER-3): results from two phase 3 randomised trials. Lancet (2015) 386(9993):541–51. doi: 10.1016/s0140-6736(15)60125-8 26072109

[35] Azevedo A Torres T. Clinical efficacy and safety of ixekizumab for treatment of psoriasis. Actas Dermosifiliogr (2017) 108(4):305–14. doi: 10.1016/j.ad.2016.09.021 27887675

[36] Gooderham MJ Papp KA Lynde CW. Shifting the focus - the primary role of IL-23 in psoriasis and other inflammatory disorders. J Eur Acad Dermatol Venereol (2018) 32(7):1111–9. doi: 10.1111/jdv.14868 PMC6033004 29438576

[37] Sofen H Smith S Matheson RT Leonardi CL Calderon C Brodmerkel C. Guselkumab (an IL-23-specific mAb) demonstrates clinical and molecular response in patients with moderate-to-severe psoriasis. J Allergy Clin Immunol (2014) 133(4):1032–40. doi: 10.1016/j.jaci.2014.01.025 24679469

[38] Crowley JJ Warren RB Cather JC. Safety of selective IL-23p19 inhibitors for the treatment of psoriasis. J Eur Acad Dermatol Venereol (2019) 33(9):1676–84. doi: 10.1111/jdv.15653 PMC6771721 31054215

