# Supplementary material for: Comparation of time-course, dose-effect, influencing factors and adverse events of biologics in the treatment of adults with moderate to severe plaque psoriasis
Source: Front Immunol. 2023 May 25;14:1151977. doi: 10.3389/fimmu.2023.1151977 (PMC10248012; doi:10.3389/fimmu.2023.1151977)
Supplement: Supplementary file 1 [file DataSheet_1.pdf]

Supplementary Materials

Comparation of Time-Course, Dose-Effect, Influencing Factors and Adverse Events of Biologics in the Treatment of Adult Patients with Moderate to Severe Plaque Psoriasis

Weiqi Gao[1,2,3], Boran Yu[4], Ya Yan[5], Libo Zhao[1, 6, 7], Rongsheng Zhao[*,1, 6, 7]

[1] Department of Pharmacy, Peking University Third Hospital, Beijing 100191, China;
[2] Shanxi Bethune Hospital, Shanxi Academy of Medical Sciences, Tongji Shanxi Hospital, Third Hospital of Shanxi Medical University, Taiyuan, 030032, China;
[3] Tongji Hospital, Tongji Medical College, Huazhong University of Science and Technology, Wuhan, 430030, China;
[4] Department of pharmacy, Beijing Children's Hospital, Capital Medical University, National Center for Children's Health, Beijing, 100045, China;
[5] School of Pharmacy, Shanxi Medical University, Taiyuan, 030032, China.
[6] Institute for Drug Evaluation, Peking University Health Science Center, Beijing 100191, China;
[7] Therapeutic Drug Monitoring and Clinical Toxicology Center, Peking University, Beijing 100191, China;

*** Correspondence:**

Prof. Rongsheng Zhao, zhaorongsheng@bjmu.edu.cn

Department of Pharmacy, Peking University Third Hospital, 49 North Garden Road, Haidian District, Beijing, 100191, China.

Tel: +86 10 82265810

Table of Contents

References and Time Points of Included Studies 03

Risk of Bias Assessment 12

Model Development 14

Final Models 17

Model fitted time-course plots of response rate for PASI75 17

Model fitted time-course plots of response rate for PASI90 20

Model fitted time-course plots of response rate for PASI100 23

Diagnostic Plots 25

Network plot 26

Model Code 27

Code for PASI75 Model 27

Code for PASI90 Model 30

Code for PASI100 Model 33

References (Included 49 articles) 36

References and Time Points of Included Studies

Supplementary Table S1. Time points for studies included in the model-based meta-analysis.

No.	Study	Patients	Drug	Regimen	Time points (week)		
					PASI75	PASI90	PASI100
1	K.A. Papp(1) 2016	AMAGINE-1 661	Brodalumab	140mg; 210mg	2, 4, 6, 8, 10, 12	2, 4, 6, 8, 10, 12	4, 6, 8, 10, 12
2	Lebwohl M(2) 2015	AMAGINE-2 1831	Brodalumab Ustekinumab	Bro:140mg/210mg Ust:45mg weight ≤100 kg 90 mg weight >100 kg	2, 4, 6, 8, 10, 12	2, 4, 6, 8, 10, 12	4, 6, 8, 10, 12
3	Lebwohl M(2) 2015	AMAGINE-3 1881	Brodalumab Ustekinumab	Bro:140mg/210mg Ust:45mg weight ≤100 kg 90 mg weight >100 kg	2, 4, 6, 8, 10, 12	2, 4, 6, 8, 10, 12	4, 6, 8, 10, 12
4	Nakagawa(3) 2016	151	Brodalumab	70mg; 140mg; 210mg	2, 4, 6, 8, 10, 12	2, 4, 6, 8, 10, 12	4, 6, 8, 10, 12
5	Seong Jun Seo(4)	62	Brodalumab	210mg	2, 4, 6, 8, 10, 12	2, 4, 6, 8, 10, 12	4, 6, 8, 10, 12

	2021						
6	Kim A.Papp(5) 2018	250	Bimekizumab	64mg; (LD320mg)160mg; 160mg; 320mg; 480mg,	4, 8, 12	4, 8, 12	4, 8, 12
7	Kristian Reich(6) 2021	567	Bimekizumab Ustekinumab	Bim: 320mg; Ust:45mg weight ≤100 kg 90 mg weight >100 kg	4, 8, 12, 16	4, 8, 12, 16	4, 8, 12, 16
8	Gordon(7) 2021	435	Bimekizumab	320mg	2, 4, 8, 12, 16	2, 4, 8, 12, 16	2, 4, 8, 12, 16
9	Gottlieb(8) 2018	CIMPASI-1 234	Certolizumab	400mg 0, 2, 4w followed by 200mg; 400mg	4, 8, 12, 16	4, 8, 12, 16	/
10	Gottlieb(8) 2018	CIMPASI-2 226	Certolizumab	400mg 0, 2, 4w followed by 200mg; 400mg	4, 8, 12, 16	4, 8, 12, 16	/
11	Mark Lebwohl(9) 2018	559	Certolizumab Etanercept	Cer: 400mg 0, 2, 4w followed by 200mg; 400mg	4, 8, 12, 16	4, 8, 12, 16	/

				Eta: 50mg			
12	Umezawa(10) 2021	127	Certolizumab	400mg 0, 2, 4w followed by 200mg; 400mg	4, 8, 12, 16	4, 8, 12, 16	/
13	OHTSUKI(11) 2018	192	Guselkumab	50mg; 100mg	2, 4, 8, 12, 16	2, 4, 8, 12, 16	4, 8, 12, 16
14	Gordon(12) 2015	293	Guselkumab Adalimumab	Gus: 5mg; 15mg; 50mg; 100mg; 200mg; Ada:(LD80mg)40mg	4, 8, 12, 16	4, 8, 12, 16	4, 8, 12, 16
15	K.B. Gordon(13) 2016	UNCOVER-1 1296	Ixekizumab	(LD160mg)80mg, q2w; (LD160mg)80mg, q4w	2, 4, 8, 12	2, 4, 8, 12	4, 8, 12
16	Griffiths(14) 2015	UNCOVER-2 1224	Etanercept Ixekizumab	Eta: 50mg; Ixe: (LD160mg)80mg, q2w; (LD160mg)80mg, q4w	2, 4, 8, 12	2, 4, 8, 12	2, 4, 8, 12
17	Kristian Reich(15) 2018	UNCOVER-3	Etanercept Ixekizumab	Eta: 50mg; Ixe: (LD160mg)80mg,	2, 4, 8, 12	2, 4, 8, 12	2, 4, 8, 12

		1346		q2w; (LD160mg)80mg, q4w			
18	Leonardi(16) 2012	141	Ixekizumab	10mg; 25mg; 75mg; 150mg	2, 4, 6, 8, 12	2, 4, 6, 8, 12	4, 6, 8, 12
19	K. Reich(17) 2019	205	Mirikizumab	30mg; 100mg; 300mg	4, 8, 12, 16	4, 8, 12, 16	4, 8, 12, 16
20	OHTSUKI(18) 2019	171	Risankizumab	75mg; 150mg	16	4, 8, 12, 16	4, 8, 12, 16
21	Blauvelt(19) 2020	507	Risankizumab	150mg	4, 8, 12, 16	4, 8, 12, 16	4, 8, 12, 16
22	Gordon(20) 2018	UltlMMa-1 506	Risankizumab Ustekinumab	Ris:150mg Ust:45mg weight ≤100 kg 90 mg weight >100 kg	12	4, 8, 12, 16	4, 8, 12, 16
23	Gordon(20) 2018	UltlMMa-2 491	Risankizumab Ustekinumab	Ris:150mg Ust:45mg weight ≤100 kg	12	4, 8, 12, 16	4, 8, 12, 16

				90 mg weight >100 kg			
24	Sigurgeirsson(21) 2021	214	Secukinumab	2*150mg; 300mg	2, 3, 4, 8, 12	2, 3, 4, 8, 12	4, 8, 12
25	Sigurgeirsson(22) 2022	122	Secukinumab	2*150mg; 300mg	4, 12	4, 12	4,12
26	P. Rich(23) 2012	271	Secukinumab	75m; 150mg; 300mg	2, 4, 8, 12	2, 4, 8, 12	/
27	Langley(24) 2014	ERASURE 734	Secukinumab	150mg; 300mg	2, 3, 4, 8, 12	4, 8, 12	4, 8, 12
28	Lin Cai(25) 2020	441	Secukinumab	150mg; 300mg	4, 8, 12	4, 8, 12	/
29	K.A. Papp(26) 2012	125	Secukinumab	25mg, once or q4w; 75mg; 150mg	2, 4, 8, 12	8, 12, 16	/
30	A. Blauvelt(27) 2015	177	Secukinumab	150mg; 300mg	2, 3, 4, 8, 12	3, 4, 8, 12	4, 8, 12
31	C. Paul(28) 2015	181	Secukinumab	150mg; 300mg	2, 3, 4, 8, 12	2, 3, 4, 8, 12	4, 8, 12

32	Langley(24) 2014	FIXTURE 1297	Secukinumab Etanercept	Sec:150mg; 300mg; Eta:50mg	4, 8, 12	4, 8, 12	4, 8, 12
33	Kim A Papp(29) 2021	313	Sonelokimab Secukinumab	Son:30mg; 60mg; 120mg 0,2,4,8w; 120mg q2w; Sec:300mg	12	4, 8, 12	4, 8, 12
34	K. Papp(30) 2015	350	Tildrakizuma b	5mg; 25mg; 100mg; 200mg	2, 4, 6, 8, 12, 16	16	16
35	Kristian Reich(31) 2017	reSURFACE 1 772	Tildrakizuma b	100mg; 200mg	4, 8, 12	12	12
36	Kristian Reich(31) 2017	reSURFACE 2 1090	Tildrakizuma b Etanercept	Til:100mg; 200mg Eta:50mg	4, 8, 12	12	12
37	Leonardi(32) 2008	PHOENIX1 766	Ustekinumab	45mg; 90mg	2, 4, 8, 12	2, 4, 8, 12	12
38	Kim A Papp(33) 2008	PHOENIX2 1230	Ustekinumab	45mg; 90mg	2, 4, 8, 12	4, 8, 12	12

39	IGARASHI(34) 2012	157	Ustekinumab	45mg; 90mg	2, 4, 8, 12	2, 4, 8, 12	/
40	Tsen-Fang Tsai(35) 2011	119	Ustekinumab	45mg weight ≤100 kg 90 mg weight >100 kg	4, 8, 12	12	12
41	Zhang(36) 2022	187	Vunakizumab	40mg; 80mg; 160; 240mg	2, 4, 6, 8, 12, 16, 20	2, 4, 6, 8, 12, 16, 20	4, 8, 12, 16, 20
42	Andrew Blauvelt(37) 2017	VOYAGE1 837	Guselkumab Adalimumab	Gus:100mg; Ada: (LD80mg)40mg	2, 4, 8, 12, 16	2, 4, 8, 12, 16	4, 8, 12, 16
43	Kristian Reich(38) 2017	VOYAGE2 992	Guselkumab Adalimumab	Gus:100mg; Ada: (LD80mg)40mg	2, 4, 8, 12, 16	2, 4, 8, 12, 16	4, 8, 12, 16
44	Hideshi Torii(39) 2010	54	Infliximab	5mg/kg	2, 6, 10, 14	2, 6, 10, 14	/
45	Alice B. Gottlieb(40) 2004	249	Infliximab	3mg/kg; 5mg/kg	2, 4, 6, 8, 10	10	/
46	Kristian Reich(41)	353	Infliximab	5mg/kg	2, 6, 10, 14,	2, 6, 10, 14,	/

	2005				22,24	22,24	
47	Krupashankar(42) 2014	223	Itolizumab	0.4mg/kg 0, 1, 2, 3, 4w then 1.6mg/kg q2w; 1.6mg/kg q2w	2, 3, 4, 6, 8,10, 12	/	/
48	L. Cai(43) 2016	425	Adalimumab	Ada: (LD80mg)40mg	3, 7, 12	3, 7, 12	3, 7, 12
49	ASAHINA(44) 2010	169	Adalimumab	40mg; 80mg; 80mg 0w followed by 40mg	4, 8, 12, 16	4, 8, 12, 16	/
50	Alan Menter(45) 2008	1212	Adalimumab	80mg 0w followed by 40mg	4, 8, 12, 16	4, 12, 16	4, 12, 16
51	A.B. Gottlieb(46) 2011	347	Etanercept Briakinumab	Eta:50mg Bri:200mg 0,4w_100mg 8w	4, 8, 12	4, 8, 12	4, 8, 12
52	B.E. Strober(47) 2011	350	Etanercept Briakinumab	Eta:50mg Bri:200mg 0,4w_100mg 8w	4, 8, 12	4, 8, 12	4, 8, 12
53	Alice B.	112	Etanercept	25mg	2, 4, 8, 12, 16,	12, 24	/

	Gottlieb(48) 2003				20, 24		
54	K.A.Papp(49) 2005	583	Etanercept	25mg; 50mg	2, 4, 8, 12	12	/

PASI, Psoriasis Area and Severity Index score; PASI75, PASI90 and PASI100, the proportion of patients whose PASI decreased by ≥75%, 90% and 100% compared with baseline, respectively; LD, Loading dose; w, week; q2w, once every 2 weeks; q4w, once every 4 weeks.

Risk of Bias Assessment

The risk of bias in the included studies was independently assessed by Weiqi Gao, Boran Yu and Ya Yan using the Cochrane Risk of Bias Tool. The evaluation items included random sequence generation, allocation concealment, blinding of participants and personnel, blinding in the outcome assessment, incomplete outcome data, selective reporting, and other biases. Disagreements were resolved through discussion with a third investigator (Libo Zhao).

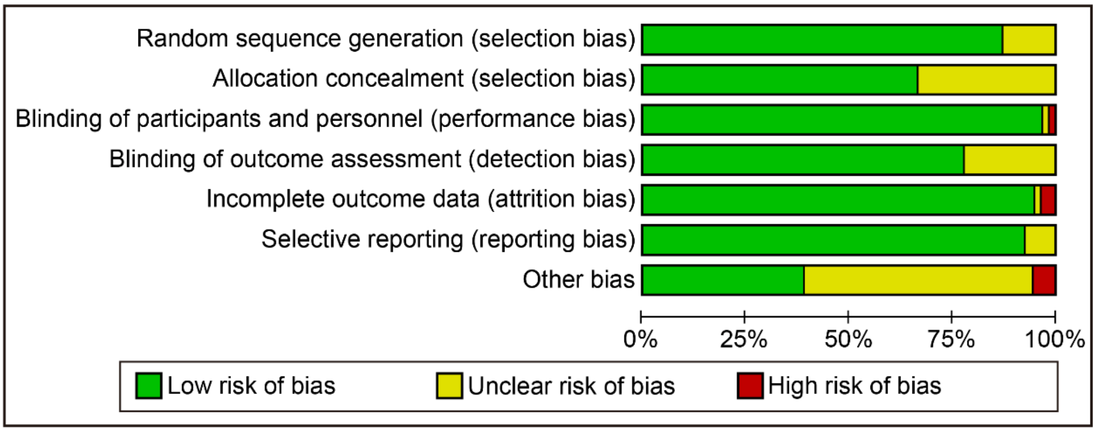

Supplementary Figure S1. Risk of bias assessment. Overall risk of bias, using Cochrane's risk of bias assessment tool.

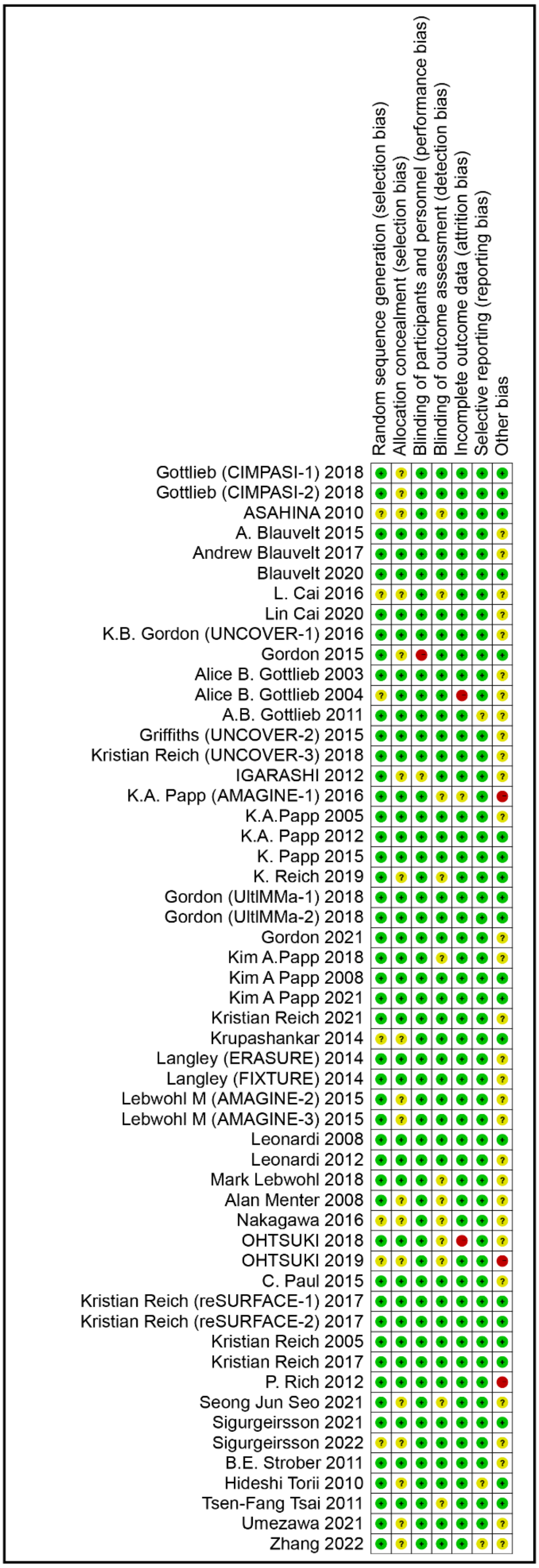

Supplementary Figure S2. Risk of bias assessment. Study-level risk of bias, using Cochrane's risk of bias assessment tool.

Model Development

1.Development of basic model

After a graphical exploration of the data, the longitudinal model of PASI75, PASI90 and PASI100 were characterized using a hierarchical regression model with the maximum likelihood estimation method. In this study, we assumed that the efficacy of the drug group consists of the placebo response and pure drug efficacy. Here, pure drug efficacy is the relative efficacy of the drug, which is the efficacy after subtracting the corresponding placebo response. The model could be commonly described as follows:

$$E_{i,j,t} = E_{placebo,i,t} + E_{drug,i,j,t} \qquad (1)$$

$$E_{drug,i,j,t} = function(drug,\ dose,\ regimen,\ time,\ \theta,\ X_{ij}) \qquad (2)$$

In formula 1, $E_{i,j,t}$ represents the efficacy in the jth treatment arm of the ith trial at t time, which is the sum of $E_{placebo,i,t}$ (the placebo effects of the ith trial at t time) and $E_{drug,i,j,t}$ (the drug effects in the jth treatment arm of the ith trial at t time). For outcomes measured as probabilities, a logit translation was performed to restrict the treatment effect to a range of 0–1. $E_{drug,i,j,t}$ in formula 2 is a function dependent on the type of drug, dose, regimen, time, fixed-effect model parameters θ, and covariates X.

2.Development of the time course relationship part in model

At first, the drug effects were set not to alter over time. Then, during model development, if the model fit improved, a time variable was added to create a non-linear model to describe the time-varying drug effects. The formula was listed as follows:

$$E_{drug} = E_{\max drug} \cdot (1\text{-}e^{-k \cdot time}) \qquad (3)$$

In formula 3, $E_{\max drug}$ represents the maximum efficacy of each treatment and k represents the rate constant describing the onset of the drug effect.

3.Development of dose-response relationship part in model

During the development of the model, the maximum efficacy of each treatment was initially incorporated to be constant over dose and described by a scaling factor, E_{max}. Then, the parameter E_{max} was separated into several parameters that match different doses, routes, and regimens of a drug. For drugs with a dose range, a dose-response relationship was estimated by the simple fixed-effect, linear, E_{max} and log-linear model. Functions of these models are listed as follows:

Simple fixed-effect model: $f = E_0 + E_{max}$ (4)

linear model: $f = E_0 + \delta \cdot Dose$ (5)

E_{max} model: $f = E_0 + \frac{Emaxdrug \cdot Dose}{ED50 + Dose}$ (6)

log-linear model: $f = E_0 + (E_{maxdrug} + \beta \cdot \log(Dose - constant))$ (7)

In which, E_0 represented the placebo efficacy, while δ was the slope parameter of linear model. $E_{maxdrug}$ represented the maximum efficacy of the drug. β was the slope parameter of log-linear model.

4.Model for PASI75, PASI90 and PASI100

$$N_{effect\ i,j,t} \sim binomial\ (P_{i,j,t}, N_{i,j}) \quad (8)$$

$$P_{i,j,t} = g\ (E_{placebo,i,t} + E_{drug\ i,j,t}) \quad (9)$$

For ratio outcomes, efficacy of a treatment arm was considered as $P_{i,j,t}$ (the probability of patients achieving endpoints at t time in jth treatment arm of ith trial), from a binomial distribution of $N_{effect\ i,j,t}$ (the number of patients achieving endpoint at t time in jth treatment arm of ith trial) with probability ($P_{i,j,t}$) and sample size ($N_{i,j}$). g was a logit translate of the efficacy of treatment, which was a sum of $E_{placebo,i,t}$ (placebo effect of ith trial at t time) and $E_{drug\ i,j,t}$ (drug effect of jth treatment arm in ith trial at t

time), to limit the probability to a range of 0-1.

For PASI75, PASI90, PASI100, the weight was based on the standard error of observed values and was generated by the following equation (formula 10) with *P* and *N*, avoiding the possible deviations in the final model caused by the extreme outcome values, and ensuring that more larger sample size outcomes had a greater impact.

$$Weight = \sqrt{\frac{P \cdot (1-P)}{N}} \qquad (10)$$

5.Covariate

Baseline characteristics, including percentage of male, age (year), weight (kg), disease duration (year), percentage of prior biological therapy and baseline PASI, were set as the covariates in the model. Covariates were investigated for their possible impact on the treatment efficacies with the following equation (formula 11), where θ was the parameter quantifying the covariate effects.

$$\text{Effect}_{\text{Covariate}} = \frac{\text{Covariate}\theta}{\text{mean(Covariate)}} \qquad (11)$$

Model development and iteration were based on the data and guided by successful convergence of the minimization routine. Model selection was based on the Akaike information criterion and the log-likelihood ratio at an acceptance *p*-value of 0.05.

Final Models

1.Model fitted time-course plots of response rate for PASI75

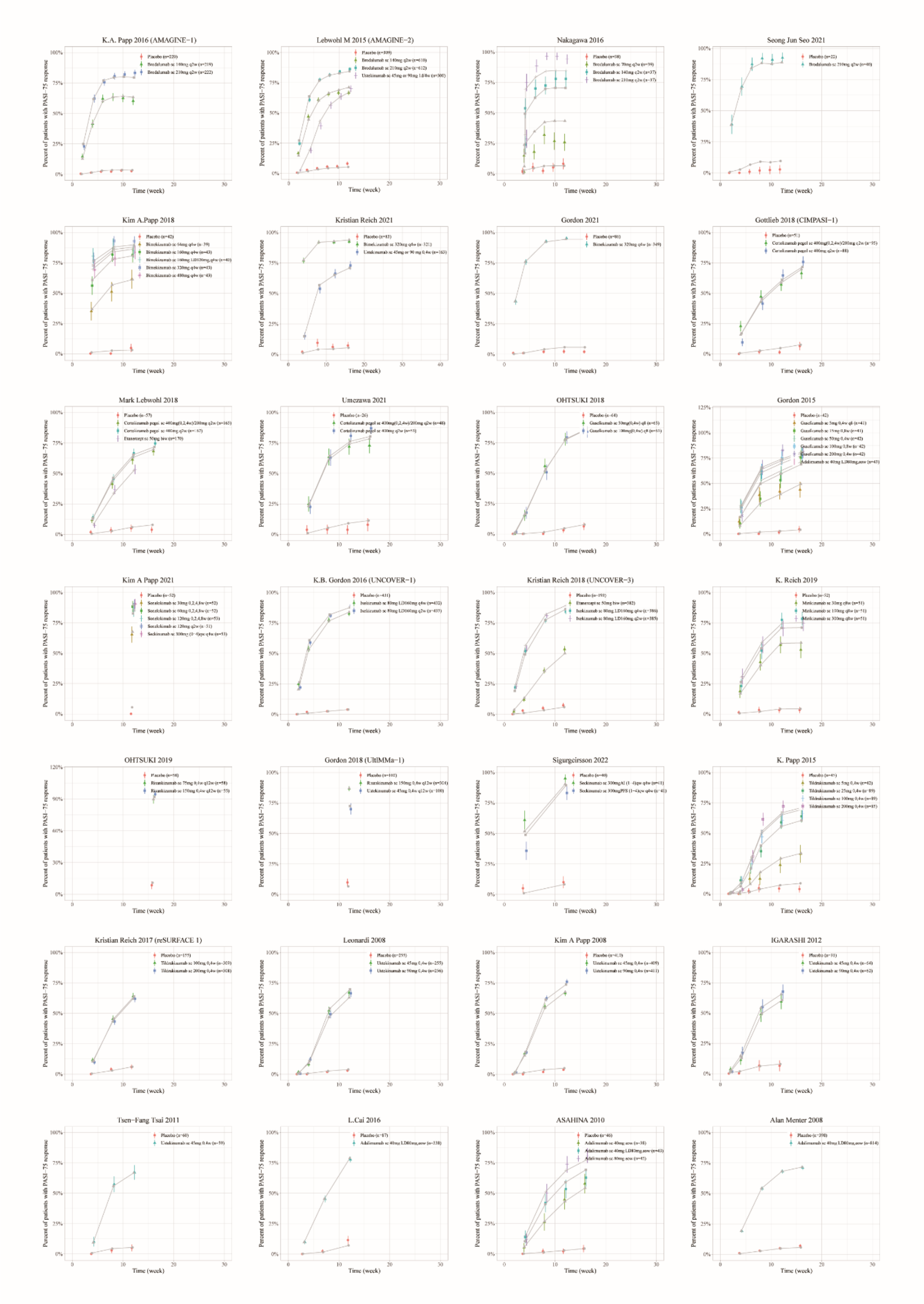

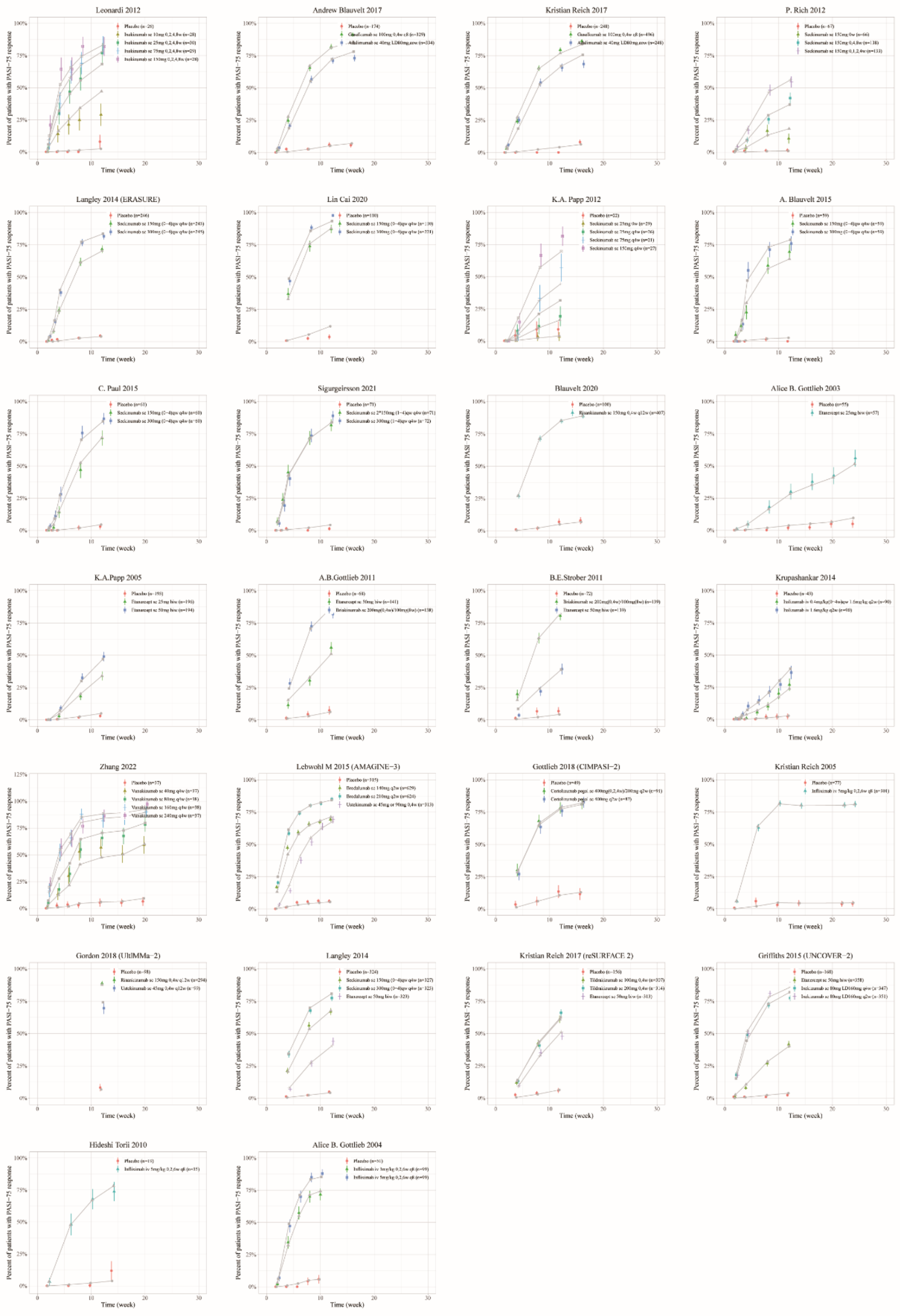

Supplementary Figure S3. Model fitted time-course plots of response rate for PASI75. Color symbols and vertical bars are observed mean and calculated weight of time points; gray symbols and lines are the model predictions. PASI, Psoriasis Area and Severity

Index score; PASI75, the proportion of patients achieving ≥75% reduction from baseline PASI score; s.c., subcutaneous injection; i.v., intravenous injection; w, week; eow, every other week; biw, twice weekly; q2w, once every 2 weeks; q4w, once every 4 weeks.

2.Model fitted time-course plots of response rate for PASI90

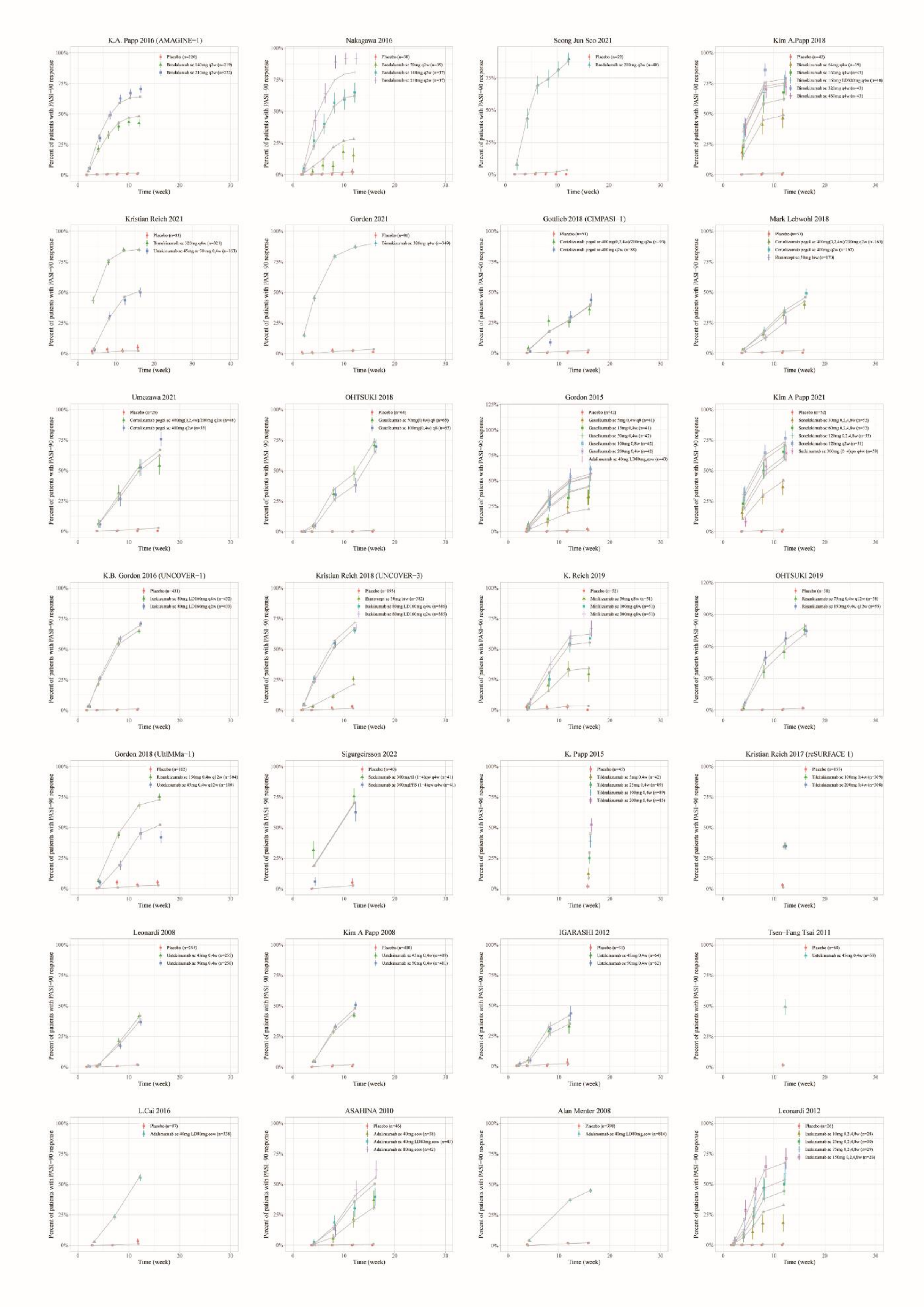

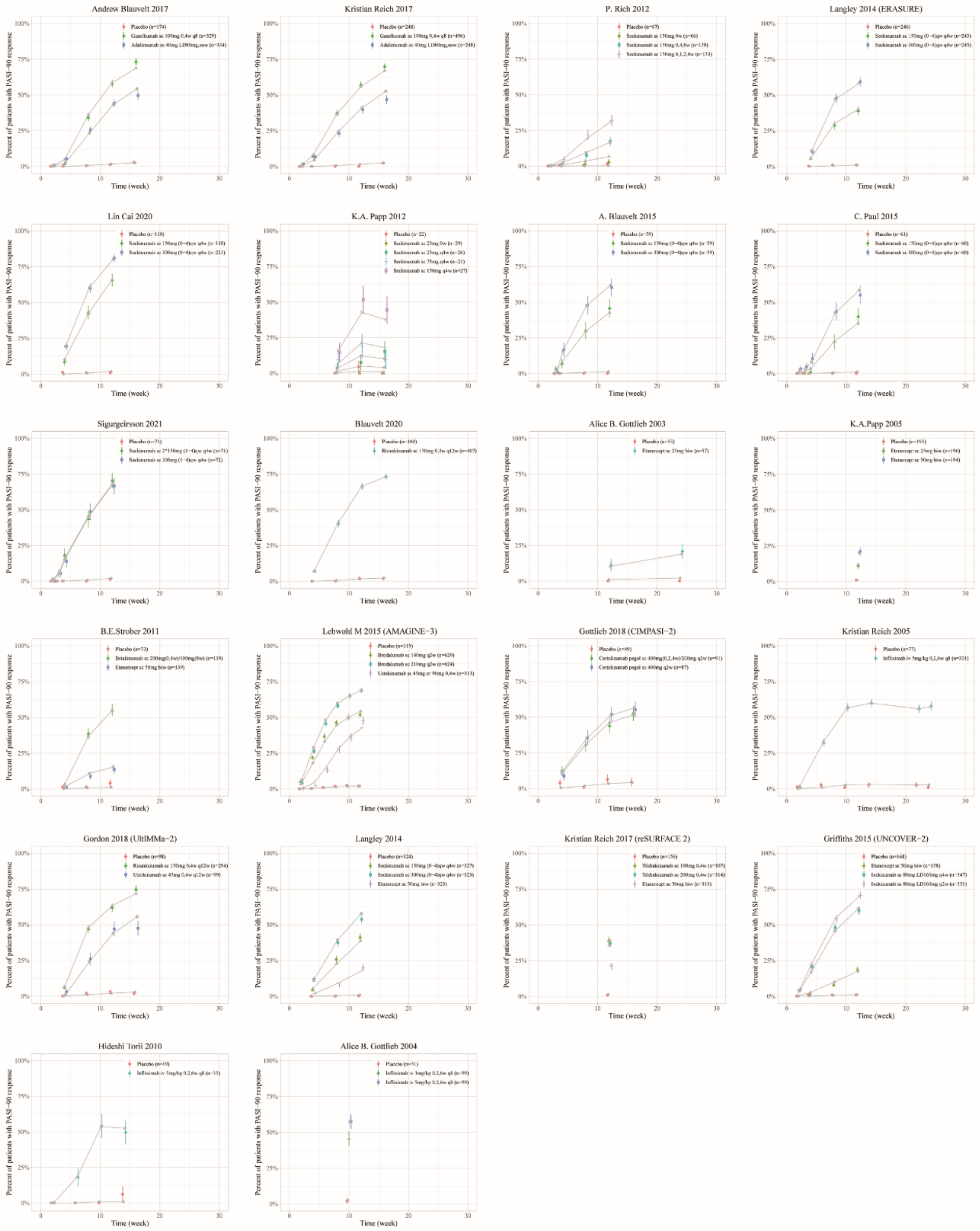

Supplementary Figure S4. Model fitted time-course plots of response rate for PASI90. Color symbols and vertical bars are observed mean and calculated weight of time points; gray symbols and lines are the model predictions. PASI, Psoriasis Area and Severity Index score; PASI90, the proportion of patients achieving ≥ 90% reduction from

baseline PASI score; s.c., subcutaneous injection; i.v., intravenous injection; w, week; eow, every other week; biw, twice weekly; q2w, once every 2 weeks; q4w, once every 4 weeks.

3.Model fitted time-course plots of response rate for PASI100

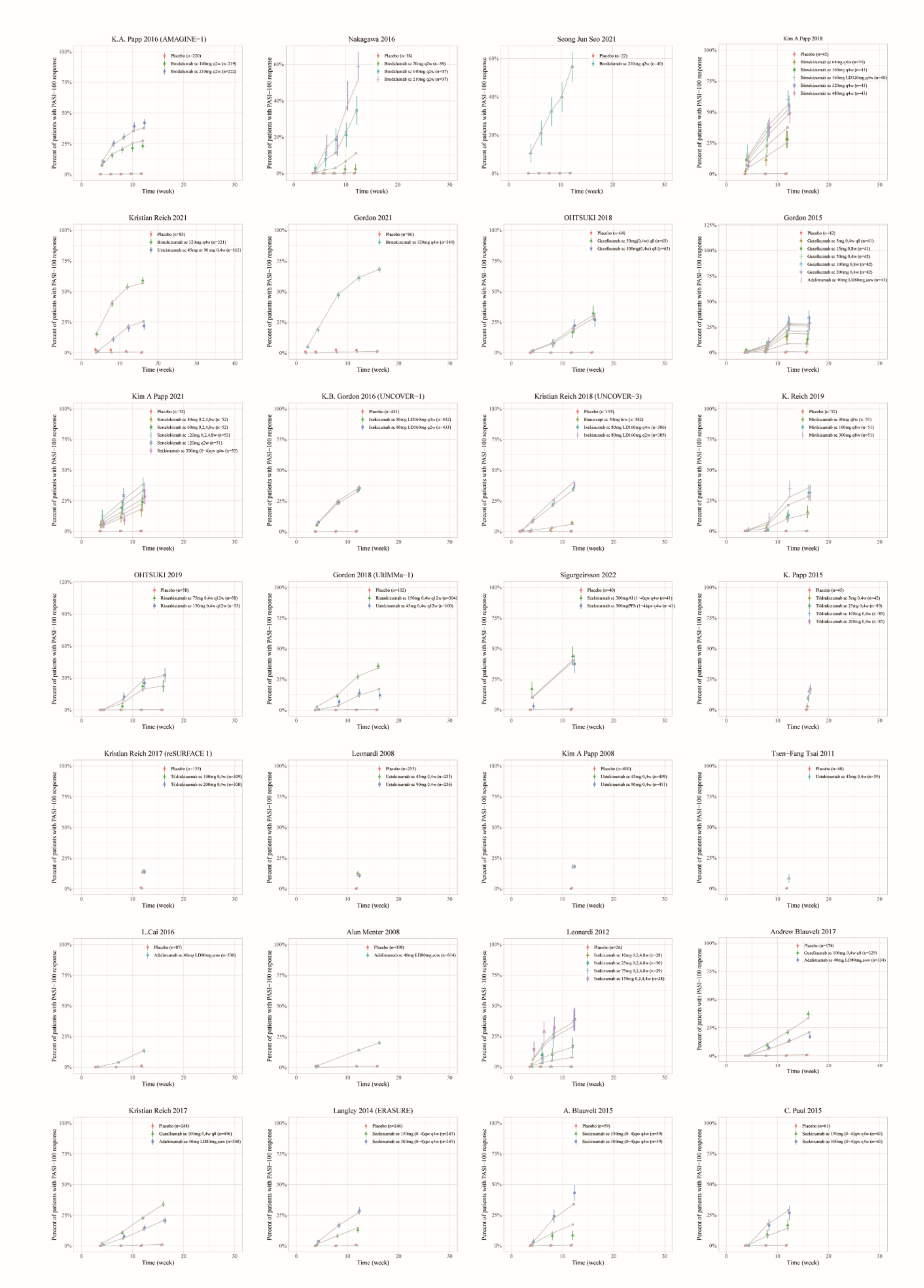

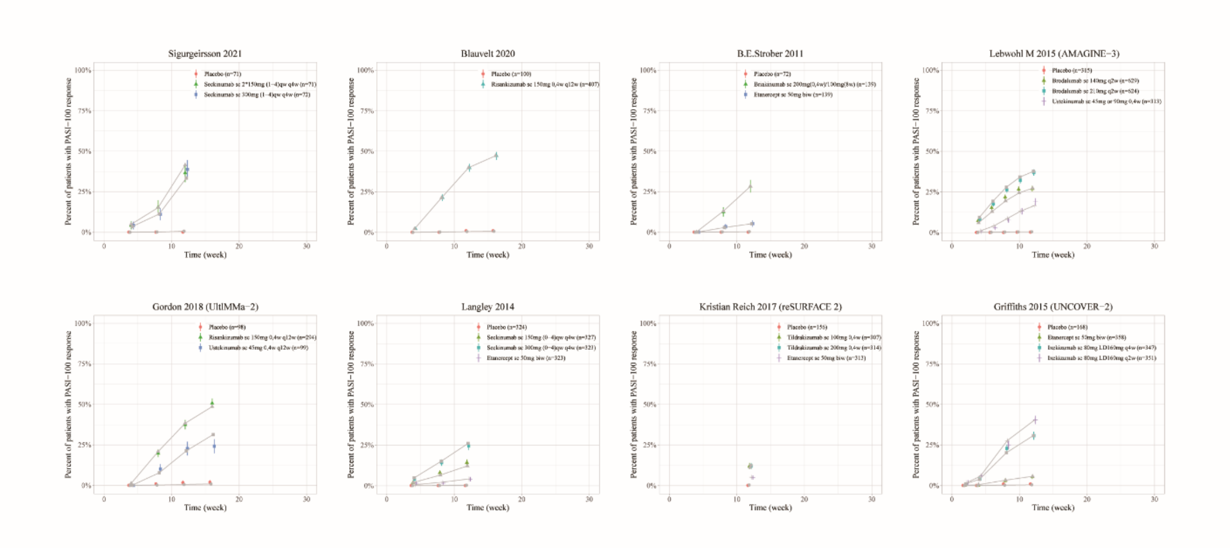

Supplementary Figure S5. Model fitted time-course plots of response rate for PASI100. Color symbols and vertical bars are observed mean and calculated weight of time points; gray symbols and lines are the model predictions. PASI, Psoriasis Area and Severity Index score; PASI100, the proportion of patients achieving ≥ 100% reduction from baseline PASI score; s.c., subcutaneous injection; i.v., intravenous injection; w, week; eow, every other week; biw, twice weekly; q2w, once every 2 weeks; q4w, once every 4 weeks.

Diagnostic Plots

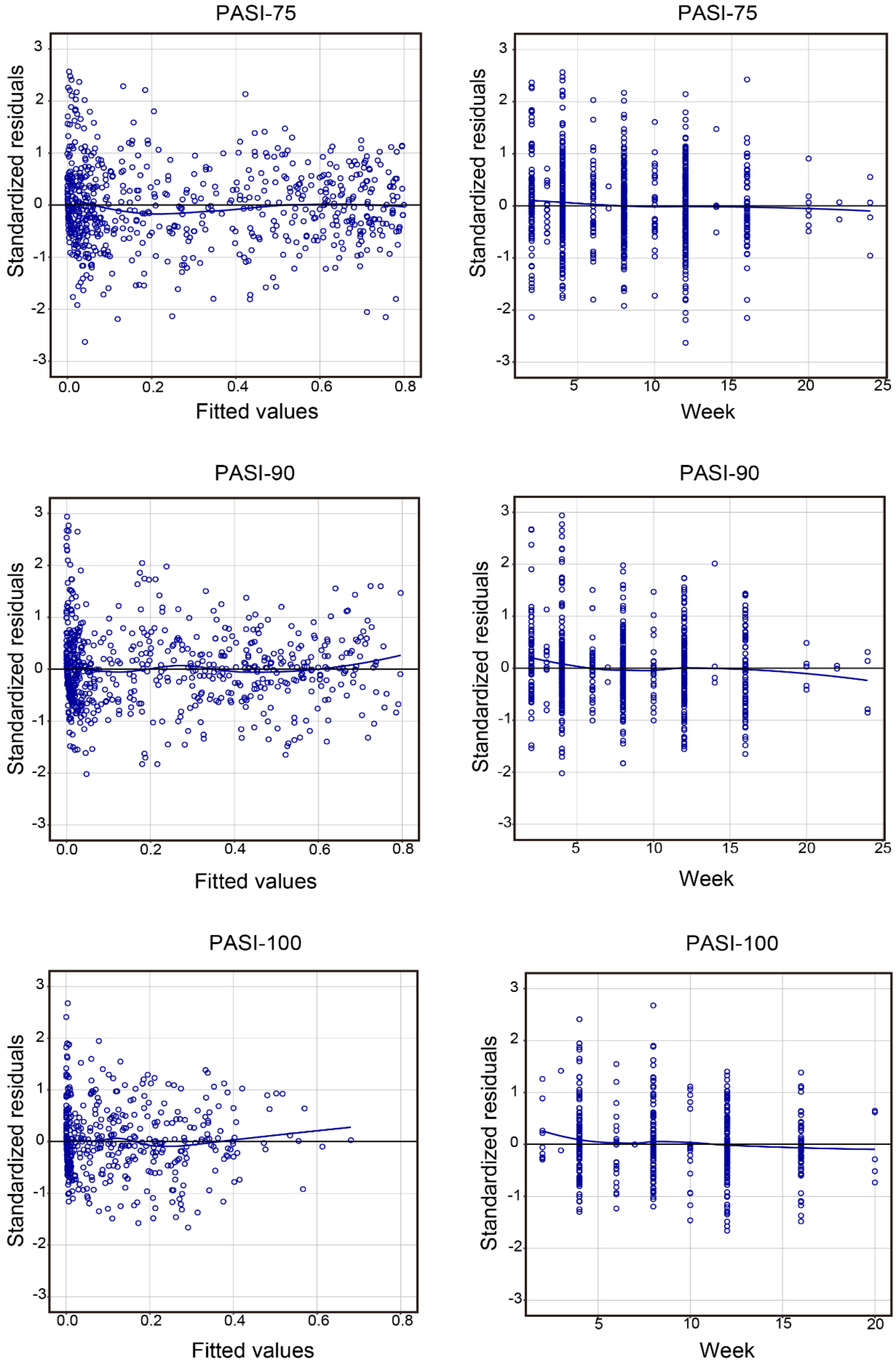

Supplementary Figure S6. Diagnostic Plots

Network plot

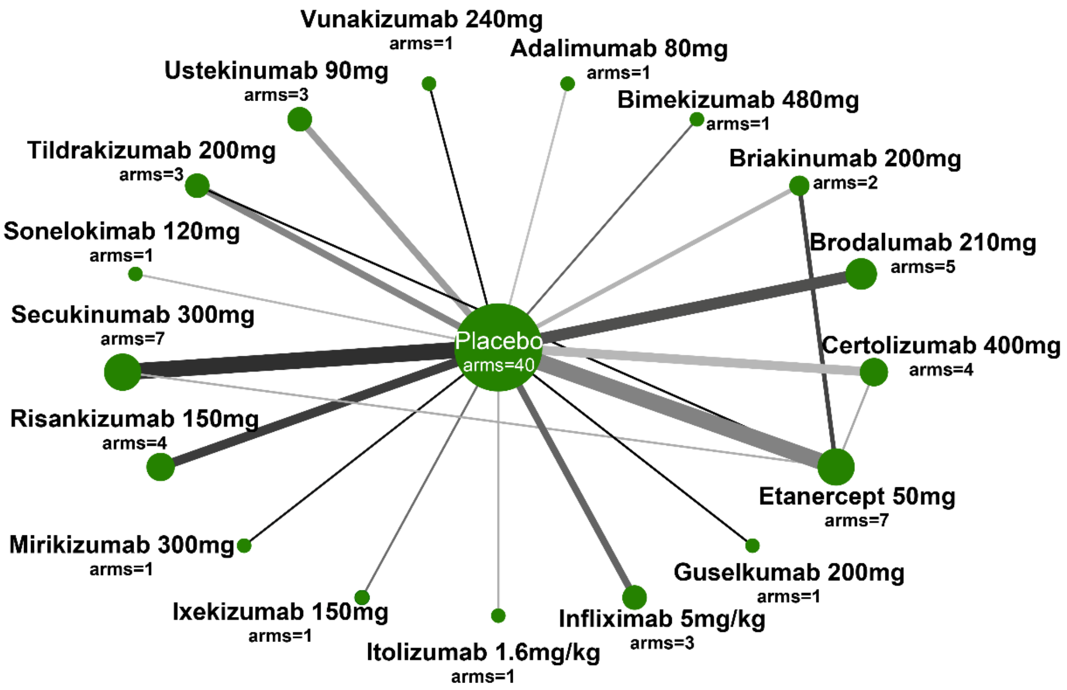

Supplementary Figure S7. Network plot presenting the trial data contributing evidence comparing Incidence rate of adverse events. Width of the lines is proportional to the number of arms comparing every pair of treatments, size of every circle is proportional to the number of randomly assigned participants.

Model Code

Code for PASI75 model

```
pasi75.27 <-
function(trialno,armno,week,CLASS,FLAG,DOSE,groupweek,methodFlag,
PASI75,no,tp1,male,age,we,dur,Priorb,baline,Regimen,eo,embro,emust,embim,emeta,
embri,emada,emcer,emgus,eminf,emito,emixe,emmir,emris,emsec,emson,emtil,emvu
n,k,ledbro,ledgus,ledbim,ledixe,ledinf,ledvun,ledito,ledsec,ledtil,ledada,ledeta,ledmir,
ledust,sadc,sadr,sads,ePriorb)
{
  emrel = 1
  emtime = 1*(CLASS!=2) + (1-exp(-exp(k)*week))*(CLASS==2)
  emrel = emrel*(Priorb/mean(All75$Priorb,na.rm = T))**ePriorb
  emrel = emrel*emtime
  efbro = 0 + ((embro*DOSE)/(DOSE+ledbro))*(FLAG==2)
  efbro = efbro*emrel
  efust = 0 + ((emust*DOSE)/(DOSE+ledust))*(FLAG==3)
  efust = efust*emrel
  efbim = 0 + ((embim*DOSE)/(DOSE+ledbim))*(FLAG==4)
  efbim = efbim*emrel
  efeta = 0 + ((emeta*DOSE)/(DOSE+ledeta))*(FLAG==5)
  efeta = efeta*emrel
  efbri = 0 + embri*(FLAG==6)
```

efbri = efbri*emrel

efada = 0 + ((emada*DOSE)/(DOSE+ledada))*(FLAG==7)

efada = efada*emrel

efcer = 0 + (emcer+sadc*log(abs(DOSE-20)))*(FLAG==8)

efcer = efcer*emrel

efgus = 0 + ((emgus*DOSE)/(DOSE+ledgus))*(FLAG==9)

efgus = efgus*emrel

efinf = 0 + ((eminf*DOSE)/(DOSE+ledinf))*(FLAG==10)

efinf = efinf*emrel

efito = 0 + ((emito*DOSE)/(DOSE+ledito))*(FLAG==11)

efito = efito*emrel

efixe = 0 + ((emixe*DOSE)/(DOSE+ledixe))*(FLAG==12)

efixe = efixe*emrel

efmir = 0 + ((emmir*DOSE)/(DOSE+ledmir))*(FLAG==13)

efmir = efmir*emrel

efris = 0 + (emris+sadr*log(abs(DOSE-20)))*(FLAG==14)

efris = efris*emrel

efsec = 0 + ((emsec*DOSE)/(DOSE+ledsec))*(FLAG==15)

efsec = efsec*emrel

efson = 0 + (emson+sads*log(abs(DOSE-20)))*(FLAG==16)

efson = efson*emrel

eftil = 0 + ((emtil*DOSE)/(DOSE+ledtil))*(FLAG==17)

```
    eftil = eftil*emrel
    efvun = 0 + ((emvun*DOSE)/(DOSE+ledvun))*(FLAG==18)
    efvun = efvun*emrel
    emax = eo + efbro + efust + efbim + efeta + efbri + efada + efcer + efgus +
    efinf + efito + efixe + efmir + efris + efsec + efson + eftil + efvun
    yp = exp(emax)/(1+exp(emax))
    yp
}
n27 <-
gnls(PASI75~pasi75.27(trialno,armno,week,CLASS,FLAG,DOSE,groupweek,metho
dFlag,PASI75,no,tp1,male,age,we,dur,Priorb,baline,Regimen,eo,embro,emust,embim,
emeta,embri,emada,emcer,emgus,eminf,emito,emixe,emmir,emris,emsec,emson,emtil,
emvun,k,ledbro,ledgus,ledbim,ledixe,ledinf,ledvun,ledito,ledsec,ledtil,ledada,ledeta,le
dmir,ledust,sadc,sadr,sads,ePriorb),data = PASI75data,
params = list(eo~-1+I(groupweek),
embro~1,emust~1,embim~1,emeta~1,embri~1,emada~1,emcer~1,emgus~1,eminf~1,e
mito~1,emixe~1,emmir~1,emris~1,emsec~1,emson~1,emtil~1,emvun~1,k~1,ledbro~
1,ledgus~1,ledbim~1,ledixe~1,ledinf~1,ledvun~1,ledito~1,ledsec~1,ledtil~1,ledada~1
,ledeta~1,ledmir~1,ledust~1,sadc~1,sadr~1,sads~1,ePriorb~1),
start = c(coef(n26),0.57),
weights=varPower(0.5,form=~fitted(.)*(1-fitted(.))/no,fixed = 0.5),
verbose = T)
```

Code for PASI90 model

```
pasi90.28 <-
function(trialno,armno,week,CLASS,FLAG,DOSE,groupweek,methodFlag,PASI90,no,tp1,male,age,we,dur,Priorb,baline,Regimen,eo,embro,emust,embim,emeta,embri,emada,emcer,emgus,eminf,emixe,emmir,emris,emsec,emson,emtil,emvun,k,ledbro,ledgus,ledbim,ledixe,ledvun,ledsec,ledtil,ledada,ledeta,ledmir,ledust,sadc,ledinf,sadr,sads,eage,ePriorb,ewe)
{
  emrel = 1
  emtime = 1*(CLASS!=2) + (1-exp(-exp(k)*week))*(CLASS==2)
  emrel = emrel*(age/mean(All90$age,na.rm = T))**eage
  emrel = emrel*(Priorb/mean(All90$Priorb,na.rm = T))**ePriorb
  emrel = emrel*(we/mean(All90$we,na.rm = T))**ewe
  emrel = emrel*emtime
  efbro = 0 + ((embro*DOSE)/(DOSE+ledbro))*(FLAG==2)
  efbro = efbro*emrel
  efust = 0 + ((emust*DOSE)/(DOSE+ledust))*(FLAG==3)
  efust = efust*emrel
  efbim = 0 + ((embim*DOSE)/(DOSE+ledbim))*(FLAG==4)
  efbim = efbim*emrel
  efeta = 0 + ((emeta*DOSE)/(DOSE+ledeta))*(FLAG==5)
  efeta = efeta*emrel
```

```
efbri = 0 + embri*(FLAG==6)

efbri = efbri*emrel

efada = 0 + ((emada*DOSE)/(DOSE+ledada))*(FLAG==7)

efada = efada*emrel

efcer = 0 + (emcer+sadc*log(abs(DOSE-20)))*(FLAG==8)

efcer = efcer*emrel

efgus = 0 + ((emgus*DOSE)/(DOSE+ledgus))*(FLAG==9)

efgus = efgus*emrel

efinf = 0 + ((eminf*DOSE)/(DOSE+ledinf))*(FLAG==10)

efinf = efinf*emrel

efixe = 0 + ((emixe*DOSE)/(DOSE+ledixe))*(FLAG==12)

efixe = efixe*emrel

efmir = 0 + ((emmir*DOSE)/(DOSE+ledmir))*(FLAG==13)

efmir = efmir*emrel

efris = 0 + (emris+sadr*log(abs(DOSE-20)))*(FLAG==14)

efris = efris*emrel

efsec = 0 + ((emsec*DOSE)/(DOSE+ledsec))*(FLAG==15)

efsec = efsec*emrel

efson = 0 + (emson+sads*log(abs(DOSE-20)))*(FLAG==16)

efson = efson*emrel

eftil = 0 + ((emtil*DOSE)/(DOSE+ledtil))*(FLAG==17)

eftil = eftil*emrel
```

```
  efvun = 0 + ((emvun*DOSE)/(DOSE+ledvun))*(FLAG==18)
  efvun = efvun*emrel
  emax = eo + efbro + efust + efbim + efeta + efbri + efada + efcer + efgus +
  efinf + efixe + efmir + efris + efsec + efson + eftil + efvun
  yp = exp(emax)/(1+exp(emax))
  yp
}
n28 <-
gnls(PASI90~pasi90.28(trialno,armno,week,CLASS,FLAG,DOSE,groupweek,metho
dFlag,PASI90,no,tp1,male,age,we,dur,Priorb,baline,Regimen,eo,embro,emust,embim,
emeta,embri,emada,emcer,emgus,eminf,emixe,emmir,emris,emsec,emson,emtil,emvu
n,k,ledbro,ledgus,ledbim,ledixe,ledvun,ledsec,ledtil,ledada,ledeta,ledmir,ledust,sadc,l
edinf,sadr,sads,eage,ePriorb,ewe), data = PASI90data,
params = list(eo~-1+I(groupweek),
embro~1,emust~1,embim~1,emeta~1,embri~1,emada~1,emcer~1,emgus~1,eminf~1,e
mixe~1,emmir~1,emris~1,emsec~1,emson~1,emtil~1,emvun~1,k~1,ledbro~1,ledgus~
1,ledbim~1,ledixe~1,ledvun~1,ledsec~1,ledtil~1,ledada~1,ledeta~1,ledmir~1,ledust~
1,sadc~1,ledinf~1,sadr~1,sads~1,eage~1,ePriorb~1,ewe~1),
start = c(coef(n27),0.040),
weights=varPower(0.5,form=~fitted(.)*(1-fitted(.))/no,fixed = 0.5),
verbose = T
)
```

Code for PASI100 model

```
pasi100.24 <-
function(trialno,armno,week,CLASS,FLAG,DOSE,groupweek,methodFlag,PASI100,
no,tp1,Regimen,male,age,we,dur,Priorb,baline,eo,embro,emust,embim,emeta,embri,e
mada,emgus,emixe,emmir,emris,emsec,emson,emtil,emvun,k,ledbro,ledgus,ledbim,le
dixe,ledvun,ledsec,ledtil,ledmir,ledust,sadr,sads,eage,ewe,edur)
{
  emrel = 1
  emtime = 1*(CLASS!=2) + (1-exp(-exp(k)*week))*(CLASS==2)
  emrel = emrel*(age/mean(All100$age,na.rm = T))**eage
  emrel = emrel*(we/mean(All100$we,na.rm = T))**ewe
  emrel = emrel*(dur/mean(All100$dur,na.rm = T))**edur
  emrel = emrel*emtime
  efbro = 0 + ((embro*DOSE)/(DOSE+ledbro))*(FLAG==2)
  efbro = efbro*emrel
  efust = 0 + ((emust*DOSE)/(DOSE+ledust))*(FLAG==3)
  efust = efust*emrel
  efbim = 0 + ((embim*DOSE)/(DOSE+ledbim))*(FLAG==4)
  efbim = efbim*emrel
  efeta = 0 + emeta*(FLAG==5)
  efeta = efeta*emrel
  efbri = 0 + embri*(FLAG==6)
```

```
efbri = efbri*emrel

efada = 0 + emada*(FLAG==7)

efada = efada*emrel

efgus = 0 + ((emgus*DOSE)/(DOSE+ledgus))*(FLAG==9)

efgus = efgus*emrel

efixe = 0 + ((emixe*DOSE)/(DOSE+ledixe))*(FLAG==12)

efixe = efixe*emrel

efmir = 0 + ((emmir*DOSE)/(DOSE+ledmir))*(FLAG==13)

efmir = efmir*emrel

efris = 0 + (emris+sadr*log(abs(DOSE-20)))*(FLAG==14)

efris = efris*emrel

efsec = 0 + ((emsec*DOSE)/(DOSE+ledsec))*(FLAG==15)

efsec = efsec*emrel

efson = 0 + (emson+sads*log(abs(DOSE-20)))*(FLAG==16)

efson = efson*emrel

eftil = 0 + ((emtil*DOSE)/(DOSE+ledtil))*(FLAG==17)

eftil = eftil*emrel

efvun = 0 + ((emvun*DOSE)/(DOSE+ledvun))*(FLAG==18)

efvun = efvun*emrel

emax = eo + efbro + efust + efbim + efeta + efbri + efada + efgus +

efixe + efmir + efris + efsec + efson + eftil + efvun

yp = exp(emax)/(1+exp(emax))
```

```
   yp
}
n24 <-
gnls(PASI100~pasi100.24(trialno,armno,week,CLASS,FLAG,DOSE,groupweek,meth
odFlag,PASI100,no,tp1,Regimen,male,age,we,dur,Priorb,baline,eo,embro,emust,embi
m,emeta,embri,emada,emgus,emixe,emmir,emris,emsec,emson,emtil,emvun,k,ledbro,
ledgus,ledbim,ledixe,ledvun,ledsec,ledtil,ledmir,ledust,sadr,sads,eage,ewe,edur),
data = PASI100data,
params = list(eo~-1+I(groupweek),
embro~1,emust~1,embim~1,emeta~1,embri~1,emada~1,emgus~1,emixe~1,emmir~1,
emris~1,emsec~1,emson~1,emtil~1,emvun~1,k~1,ledbro~1,ledgus~1,ledbim~1,
                    ledixe~1,ledvun~1,ledsec~1,ledtil~1,ledmir~1,ledust~1,sadr~1,
                    sads~1,eage~1,ewe~1,edur~1),
start = c(coef(n23),-0.69),
weights=varPower(0.5,form=~fitted(.)*(1-fitted(.))/no,fixed = 0.5),
verbose = T
)
```

References

1. K A Papp, K Reich, C Paul, A Blauvelt, W Baran, C Bolduc, et al. A prospective phase III, randomized, double-blind, placebo-controlled study of brodalumab in patients with moderate-to-severe plaque psoriasis. *Br J Dermatol* (2016) 175(2), 273-286. doi:10.1111/bjd.14493

2. M Lebwohl, B Strober, A Menter, K Gordon, J Weglowska, L Puig, et al. Phase 3 Studies Comparing Brodalumab with Ustekinumab in Psoriasis. *N Engl J Med* (2015) 373(14), 1318-1328. doi:10.1056/NEJMoa1503824

3. H Nakagawa, H Niiro, K Ootaki. Brodalumab, a human anti-interleukin-17-receptor antibody in the treatment of Japanese patients with moderate-to-severe plaque psoriasis: Efficacy and safety results from a phase II randomized controlled study. *J Dermatol Sci* (2016) 81(1), 44-52. doi:10.1016/j.jdermsci.2015.10.009

4. S J Seo, B S Shin, J H Lee. Efficacy and safety of brodalumab in the Korean population for the treatment of moderate to severe plaque psoriasis: A randomized, phase III, double-blind, placebo-controlled study. *J Dermatol* (2021) 48(6), 807-817. doi:10.1111/1346-8138.15733

5. K A Papp, J F Merola, A B Gottlieb, C E M Griffiths, N Cross, L Peterson, et al. Dual neutralization of both interleukin 17A and interleukin 17F with bimekizumab in patients with psoriasis: Results from BE ABLE 1, a 12-week randomized, double-blinded, placebo-controlled phase 2b trial. *J Am Acad Dermatol* (2018) 79(2), 277-286.e10. doi:10.1016/j.jaad.2018.03.037

6. K Reich, K A Papp, A Blauvelt, R G Langley, A Armstrong, R B Warren, et al.

Bimekizumab versus ustekinumab for the treatment of moderate to severe plaque psoriasis (BE VIVID): efficacy and safety from a 52-week, multicentre, double-blind, active comparator and placebo controlled phase 3 trial. *Lancet* (2021) 397(10273), 487-498. doi:10.1016/s0140-6736(21)00125-2

7. K B.Gordon, P Foley, J G Krueger, A Pinter, K Reich, R Vender, et al. Bimekizumab efficacy and safety in moderate to severe plaque psoriasis (BE READY): a multicentre, double-blind, placebo-controlled, randomised withdrawal phase 3 trial. *Lancet* (2021) 397(10273), 475-486. doi:10.1016/s0140-6736(21)00126-4

8. A B Gottlieb, A Blauvelt, D Thaçi, C L Leonardi, Y Poulin, J Drew, et al. Certolizumab pegol for the treatment of chronic plaque psoriasis: Results through 48 weeks from 2 phase 3, multicenter, randomized, double-blinded, placebo-controlled studies (CIMPASI-1 and CIMPASI-2). *J Am Acad Dermatol* (2018) 79(2), 302-314.e6. doi:10.1016/j.jaad.2018.04.012

9. M Lebwohl, A Blauvelt, C Paul, H Sofen, J Węgłowska, V Piguet, et al. Certolizumab pegol for the treatment of chronic plaque psoriasis: Results through 48 weeks of a phase 3, multicenter, randomized, double-blind, etanercept- and placebo-controlled study (CIMPACT). *J Am Acad Dermatol* (2018) 79(2), 266-276.e5. doi:10.1016/j.jaad.2018.04.013

10. Y Umezawa, S Sakurai. Certolizumab Pegol for the Treatment of Moderate to Severe Plaque Psoriasis: 16-Week Results from a Phase 2/3 Japanese Study. *Dermatol Ther (Heidelb)* (2021) 11(2), 513-528. doi:10.1007/s13555-021-00494-z

11. M Ohtsuki, H Kubo. Guselkumab, an anti-interleukin-23 monoclonal antibody, for

the treatment of moderate to severe plaque-type psoriasis in Japanese patients: Efficacy and safety results from a phase 3, randomized, double-blind, placebo-controlled study. *J Dermatol* (2018) 45(9), 1053-1062. doi:10.1111/1346-8138.14504

12. K B Gordon, K C Duffin, R Bissonnette, J C Prinz, Y Wasfi, S Li, et al. A Phase 2 Trial of Guselkumab versus Adalimumab for Plaque Psoriasis. *N Engl J Med* (2015) 373(2), 136-144.doi:10.1056/NEJMoa1501646

13. K B Gordon, A Blauvelt, K A Papp, R G Langley, T Luger, M Ohtsuki, et al. Phase 3 Trials of Ixekizumab in Moderate-to-Severe Plaque Psoriasis. *N Engl J Med* (2016) 375(4), 345-356. doi:10.1056/NEJMoa1512711

14. C E Griffiths, K Reich, M Lebwohl, P van de Kerkhof, C Paul, A Menter, et al. Comparison of ixekizumab with etanercept or placebo in moderate-to-severe psoriasis (UNCOVER-2 and UNCOVER-3): results from two phase 3 randomised trials. *Lancet* (2015) 386(9993), 541-551. doi:10.1016/s0140-6736(15)60125-8

15. K Reich, K Jackson, S Ball, S Garces, L Kerr, L Chua,et al. Ixekizumab Pharmacokinetics, Anti-Drug Antibodies, and Efficacy through 60 Weeks of Treatment of Moderate to Severe Plaque Psoriasis. *J Invest Dermatol* (2018) 138(10), 2168-2173. doi:10.1016/j.jid.2018.04.019

16. C Leonardi, R Matheson, C Zachariae, G Cameron, L Li, E Edson-Heredia, et al. Anti-interleukin-17 monoclonal antibody ixekizumab in chronic plaque psoriasis. *N Engl J Med* (2012) 366(13), 1190-1199. doi:10.1056/NEJMoa1109997

17. K Reich, P Rich, C Maari, R Bissonnette. Efficacy and safety of mirikizumab (LY3074828) in the treatment of moderate-to-severe plaque psoriasis: results from a

randomized phase II study. *Br J Dermatol* (2019) 181(1), 88-95. doi:10.1111/bjd.17628

18. M Ohtsuki, H Fujita, M Watanabe, K Suzaki, M Flack, X Huang, et al. Efficacy and safety of risankizumab in Japanese patients with moderate to severe plaque psoriasis: Results from the SustaIMM phase 2/3 trial. *J Dermatol* (2019) 46(8), 686-694. doi:10.1111/1346-8138.14941

19. A Blauvelt, C L Leonardi, M Gooderham, K A Papp, S Philipp, J J Wu, et al. Efficacy and Safety of Continuous Risankizumab Therapy vs Treatment Withdrawal in Patients With Moderate to Severe Plaque Psoriasis: A Phase 3 Randomized Clinical Trial. *JAMA Dermatol* (2020) 156(6), 649-658. doi:10.1001/jamadermatol.2020.0723

20. K B Gordon, B Strober, M Lebwohl, M Augustin, A Blauvelt, Y Poulin, et al. Efficacy and safety of risankizumab in moderate-to-severe plaque psoriasis (UltIMMa-1 and UltIMMa-2): results from two double-blind, randomised, placebo-controlled and ustekinumab-controlled phase 3 trials. *Lancet* (2018) 392(10148), 650-661. doi:10.1016/s0140-6736(18)31713-6

21. B Sigurgeirsson, K Schäkel, C H Hong. Efficacy, tolerability, patient usability, and satisfaction with a 2 mL pre-filled syringe containing secukinumab 300 mg in patients with moderate to severe plaque psoriasis: results from the phase 3 randomized, double-blind, placebo-controlled ALLURE study. *J Dermatolog Treat* (2022) 33(3), 1718-1726. doi:10.1080/09546634.2021.1902925

22. B Sigurgeirsson, J Browning, S Tyring, J C Szepietowski. Secukinumab demonstrates efficacy, safety, and tolerability upon administration by 2 ml autoinjector

in adult patients with plaque psoriasis: 52-week results from MATURE, a randomized, placebo-controlled trial. *Dermatol Ther* (2022) 35(3), e15285. doi:10.1111/dth.15285

23. P Rich, B Sigurgeirsson, D Thaci, J P Ortonne, C Paul, R E Schopf, et al. Secukinumab induction and maintenance therapy in moderate-to-severe plaque psoriasis: a randomized, double-blind, placebo-controlled, phase II regimen-finding study. *Br J Dermatol* (2013) 168(2), 402-411. doi:10.1111/bjd.12112

24. R G Langley, B E Elewski, M Lebwohl, K Reich, C E Griffiths, K Papp, et al. Secukinumab in plaque psoriasis--results of two phase 3 trials. *N Engl J Med* (2014) 371(4), 326-338. doi:10.1056/NEJMoa1314258

25. L Cai, J Z Zhang, X Yao, J Gu, Q Z Liu, M Zheng, et al. Secukinumab demonstrates high efficacy and a favorable safety profile over 52 weeks in Chinese patients with moderate to severe plaque psoriasis. *Chin Med J (Engl)* (2020) 133(22), 2665-2673. doi:10.1097/cm9.0000000000001163

26. K A Papp, R G Langley, B Sigurgeirsson, M Abe, D R Baker, P Konno,et al. Efficacy and safety of secukinumab in the treatment of moderate-to-severe plaque psoriasis: a randomized, double-blind, placebo-controlled phase II dose-ranging study. *Br J Dermatol* (2013) 168(2), 412-421. doi:10.1111/bjd.12110

27. A Blauvelt, J C Prinz, A B Gottlieb, K Kingo, H Sofen, M Ruer-Mulard, et al. Secukinumab administration by pre-filled syringe: efficacy, safety and usability results from a randomized controlled trial in psoriasis (FEATURE). *Br J Dermatol* (2015) 172(2), 484-493. doi:10.1111/bjd.13348

28. C Paul, J P Lacour, L Tedremets, K Kreutzer, S Jazayeri, S Adams, et al. Efficacy,

safety and usability of secukinumab administration by autoinjector/pen in psoriasis: a randomized, controlled trial (JUNCTURE). *J Eur Acad Dermatol Venereol* (2015) 29(6), 1082-1090. doi:10.1111/jdv.12751

29. K A Papp, M A Weinberg, A Morris, K Reich. IL17A/F nanobody sonelokimab in patients with plaque psoriasis: a multicentre, randomised, placebo-controlled, phase 2b study. *Lancet* (2021) 397(10284), 1564-1575. doi:10.1016/s0140-6736(21)00440-2

30. K Papp, D Thaçi, K Reich, E Riedl, R G Langley, J G Krueger, et al. Tildrakizumab (MK-3222), an anti-interleukin-23p19 monoclonal antibody, improves psoriasis in a phase IIb randomized placebo-controlled trial. *Br J Dermatol* (2015) 173(4), 930-939. doi:10.1111/bjd.13932

31. K Reich, K A Papp, A Blauvelt, S K Tyring, R Sinclair, D Thaçi, et al. Tildrakizumab versus placebo or etanercept for chronic plaque psoriasis (reSURFACE 1 and reSURFACE 2): results from two randomised controlled, phase 3 trials. *Lancet* (2017) 390(10091), 276-288. doi:10.1016/s0140-6736(17)31279-5

32. C L Leonardi, A B Kimball, K A Papp, N Yeilding, C Guzzo, Y Wang, et al. Efficacy and safety of ustekinumab, a human interleukin-12/23 monoclonal antibody, in patients with psoriasis: 76-week results from a randomised, double-blind, placebo-controlled trial (PHOENIX 1). *Lancet* (2008) 371(9625), 1665-1674. doi:10.1016/s0140-6736(08)60725-4

33. K A Papp, R G Langley, M Lebwohl, G G Krueger, P Szapary, N Yeilding, et al. Efficacy and safety of ustekinumab, a human interleukin-12/23 monoclonal antibody, in patients with psoriasis: 52-week results from a randomised, double-blind, placebo-

controlled trial (PHOENIX 2). *Lancet* (2008)371(9625), 1675-1684. doi:10.1016/s0140-6736(08)60726-6

34. A Igarashi, T Kato, M Kato, M Song, H Nakagawa. Efficacy and safety of ustekinumab in Japanese patients with moderate-to-severe plaque-type psoriasis: long-term results from a phase 2/3 clinical trial. *J Dermatol* (2012) 39(3), 242-252. doi:10.1111/j.1346-8138.2011.01347.x

35. T F Tsai, J C Ho, M Song, P Szapary, C Guzzo, Y K Shen, et al. Efficacy and safety of ustekinumab for the treatment of moderate-to-severe psoriasis: a phase III, randomized, placebo-controlled trial in Taiwanese and Korean patients (PEARL). *J Dermatol Sci* (2011) 63(3), 154-163. doi:10.1016/j.jdermsci.2011.05.005

36. C Zhang, K Yan, Q Diao, Q Guo, H Jin, S Yang, et al. A multicenter, randomized, double-blinded, placebo-controlled, dose-ranging study evaluating the efficacy and safety of vunakizumab in patients with moderate-to-severe plaque psoriasis. *J Am Acad Dermatol* (2022) 87(1), 95-102. doi:10.1016/j.jaad.2022.01.005

37. A Blauvelt, K A Papp, C E Griffiths, B Randazzo, Y Wasfi, Y K Shen, et al. Efficacy and safety of guselkumab, an anti-interleukin-23 monoclonal antibody, compared with adalimumab for the continuous treatment of patients with moderate to severe psoriasis: Results from the phase III, double-blinded, placebo- and active comparator-controlled VOYAGE 1 trial. *J Am Acad Dermatol* (2017) 76(3), 405-417. doi:10.1016/j.jaad.2016.11.041

38. K Reich, A W Armstrong, P Foley, M Song, Y Wasfi, B Randazzo, et al. Efficacy and safety of guselkumab, an anti-interleukin-23 monoclonal antibody, compared with

adalimumab for the treatment of patients with moderate to severe psoriasis with randomized withdrawal and retreatment: Results from the phase III, double-blind, placebo- and active comparator-controlled VOYAGE 2 trial. *J Am Acad Dermatol* (2017) 76(3), 418-431. doi:10.1016/j.jaad.2016.11.042

39. H Torii, H Nakagawa. Infliximab monotherapy in Japanese patients with moderate-to-severe plaque psoriasis and psoriatic arthritis. A randomized, double-blind, placebo-controlled multicenter trial. *J Dermatol Sci* (2010) 59(1), 40-49. doi:10.1016/j.jdermsci.2010.04.014

40. A B Gottlieb, R Evans, S Li, L T Dooley, C A Guzzo, D Baker, et al. Infliximab induction therapy for patients with severe plaque-type psoriasis: a randomized, double-blind, placebo-controlled trial. *J Am Acad Dermatol* (2004) 51(4), 534-542. doi:10.1016/j.jaad.2004.02.021

41. K Reich, F O Nestle, K Papp, J P Ortonne, R Evans, C Guzzo, et al. Infliximab induction and maintenance therapy for moderate-to-severe psoriasis: a phase III, multicentre, double-blind trial. *Lancet* (2005) 366(9494), 1367-1374. doi:10.1016/s0140-6736(05)67566-6

42. D S Krupashankar, S Dogra, M Kura, A Saraswat, L Budamakuntla, T K Sumathy, et al. Efficacy and safety of itolizumab, a novel anti-CD6 monoclonal antibody, in patients with moderate to severe chronic plaque psoriasis: results of a double-blind, randomized, placebo-controlled, phase-III study. *J Am Acad Dermatol* (2014) 71(3), 484-492. doi:10.1016/j.jaad.2014.01.897

43. L Cai, J Gu, J Zheng, M Zheng, G Wang, L Y Xi, et al. Efficacy and safety of

adalimumab in Chinese patients with moderate-to-severe plaque psoriasis: results from a phase 3, randomized, placebo-controlled, double-blind study. *J Eur Acad Dermatol Venereol* (2017) 31(1), 89-95. doi:10.1111/jdv.13746

44. A Asahina, H Nakagawa, T Etoh, M Ohtsuki. Adalimumab in Japanese patients with moderate to severe chronic plaque psoriasis: efficacy and safety results from a Phase II/III randomized controlled study. *J Dermatol* (2010) 37(4), 299-310. doi:10.1111/j.1346-8138.2009.00748.x

45. A Menter, S K Tyring, K Gordon, A B Kimball, C L Leonardi, R G Langley, et al. Adalimumab therapy for moderate to severe psoriasis: A randomized, controlled phase III trial. *J Am Acad Dermatol* (2008) 58(1), 106-115. doi:10.1016/j.jaad.2007.09.010

46. A B Gottlieb, C Leonardi, F Kerdel, S Mehlis, M Olds, D A Williams. Efficacy and safety of briakinumab vs. etanercept and placebo in patients with moderate to severe chronic plaque psoriasis. *Br J Dermatol* (2011) 165(3), 652-660. doi:10.1111/j.1365-2133.2011.10418.x

47. B E Strober, J J Crowley, P S Yamauchi, M Olds, D A Williams. Efficacy and safety results from a phase III, randomized controlled trial comparing the safety and efficacy of briakinumab with etanercept and placebo in patients with moderate to severe chronic plaque psoriasis. *Br J Dermatol* (2011) 165(3), 661-668. doi:10.1111/j.1365-2133.2011.10419.x

48. A B Gottlieb, R T Matheson, N Lowe, G G. Krueger, S Kang, B S Goffe, et al. A randomized trial of etanercept as monotherapy for psoriasis. *Arch Dermatol* (2003) 139(12), 1627-1632. doi:10.1001/archderm.139.12.1627

49. K A Papp, S Tyring, M Lahfa, J Prinz, C E Griffiths, A M Nakanishi, et al. A global phase III randomized controlled trial of etanercept in psoriasis: safety, efficacy, and effect of dose reduction. *Br J Dermatol* (2005) 152(6), 1304-1312. doi:10.1111/j.1365-2133.2005.06688.x
